# Transglutaminase 3 regulates cutaneous squamous carcinoma differentiation and inhibits progression via PI3K-AKT signaling pathway-mediated Keratin 14 degradation

**DOI:** 10.1038/s41419-024-06626-5

**Published:** 2024-04-08

**Authors:** Kaili Zhou, Chenglong Wu, Wenjie Cheng, Boyuan Zhang, Ruoqu Wei, Daian Cheng, Yan Li, Yu Cao, Wenqing Zhang, Zhirong Yao, Xue Zhang

**Affiliations:** 1 Dermatology Center, Xinhua Hospital, Shanghai Jiaotong University School of Medicine, Shanghai, China; 2 Department of Dermatology, Shanghai Jiaotong University School of Medicine, Shanghai, China; 3 Institute of Dermatology, Shanghai Jiaotong University School of Medicine, Shanghai, China; 4 Department of Dermatology, Shanghai Skin Disease Hospital, Tongji University School of Medicine, Shanghai, China

**Keywords:** Oncogenesis, Tumour biomarkers, Phosphoinositol signalling

## Abstract

Cutaneous squamous carcinoma is the second most common epithelial malignancy, associated with significant morbidity, mortality, and economic burden. However, the mechanisms underlying cSCC remain poorly understood. In this study, we identified TGM3 as a novel cSCC tumor suppressor that acts via the PI3K-AKT axis. RT-qPCR, IHC and western blotting were employed to assess TGM3 levels. TGM3-overexpression/knockdown cSCC cell lines were utilized to detect TGM3’s impact on epithelial differentiation as well as tumor cell proliferation, migration, and invasion in vitro. Additionally, subcutaneous xenograft tumor models were employed to examine the effect of TGM3 knockdown on tumor growth in vivo. Finally, molecular and biochemical approaches were employed to gain insight into the tumor-suppressing mechanisms of TGM3. TGM3 expression was increased in well-differentiated cSCC tumors, whereas it was decreased in poor-differentiated cSCC tumors. Loss of TGM3 is associated with poor differentiation and a high recurrence rate in patients with cSCC. TGM3 exhibited tumor-suppressing activity by regulating cell proliferation, migration, and invasion both in vitro and in vivo. As a novel cSCC tumor differentiation marker, TGM3 expression was positively correlated with cell differentiation. In addition, our results demonstrated an interaction between TGM3 and KRT14 that aids in the degradation of KRT14. TGM3 deficiency disrupts keratinocytes differentiation, and ultimately leads to tumorigenesis. Furthermore, RNA-sequence analysis revealed that loss of TGM3 enhanced EMT via the PI3K-AKT signaling pathway. Deguelin, a PI3K-AKT inhibitor, blocked cSCC tumor growth induced by TGM3 knockdown in vivo. Taken together, TGM3 inhibits cSCC tumor growth via PI3K-AKT signaling, which could also serve as a tumor differentiation marker and a potential therapeutic target for cSCC.

**Figure:**
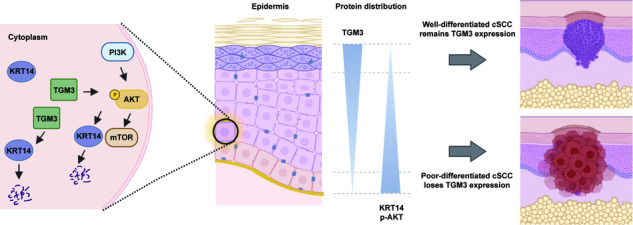
**Proposed model depicted the mechanism by which TGM3 suppress cSCC development**. TGM3 reduces the phosphorylation level of AKT and degrades KRT14. In the epithelial cell layer, TGM3 exhibits a characteristic pattern of increasing expression from bottom to top, while KRT14 and pAKT are the opposite. Loss of TGM3 leads to reduced degradation of KRT14 and activation of pAKT, disrupting keratinocyte differentiation, and eventually resulting in the occurrence of low-differentiated cSCC.

**Proposed model depicted the mechanism by which TGM3 suppress cSCC development**. TGM3 reduces the phosphorylation level of AKT and degrades KRT14. In the epithelial cell layer, TGM3 exhibits a characteristic pattern of increasing expression from bottom to top, while KRT14 and pAKT are the opposite. Loss of TGM3 leads to reduced degradation of KRT14 and activation of pAKT, disrupting keratinocyte differentiation, and eventually resulting in the occurrence of low-differentiated cSCC.

## Introduction

Cutaneous squamous carcinoma (cSCC) is the second most common epithelial malignancy, which are associated with significant morbidity, mortality, and economic burden [1–4]. Estimates of lifetime incidence of cSCC range from 6% to 11%, and this rates rising steeply in the coming years [5]. cSCC pathogenesis follows a classic tumor model, and involves multiple steps from precancerous lesion, Bowen disease to cSCC in situ and, ultimately invasion by cSCC cells [6]. In most cases, cSCC is also associated with a substantial risk of metastasis despite surgical resection [7]. Poor-differentiated tumor histology, perineural and lymphovascular invasion, and bone invasion are tumor-intrinsic risk factors for cSCC recurrence and metastasis [1]. Therefore, a comprehensive understanding of the molecular mechanisms underlying malignant differentiation and metastasis of cSCC keratinocytes is crucial for the development of effective treatment strategies.

Transglutaminase 3 (TGM3), an enzyme reported to catalyze the irreversible cross-linking of peptide-bound glutamine residues with either peptide-bound lysines or primary amines, is widely expressed in the small intestine, brain, skin, and mucosa [8]. TGM3 is mainly expressed in the suprabasal layers of the stratified squamous epithelium, and responsible for the formation and assembly of protein aggregates in the epidermis, so it is also called “epidermal TGM” [9–11]. TGM3 is associated with a various type of human cancer, including laryngeal carcinoma, hepatocellular carcinoma, esophageal carcinoma, colorectal cancer, and head and neck cancer (HNC) [12–17]. Moreover, the expression of TGM3 was downregulated in aggressive cSCC (and even absent in poor-differentiated cSCC) [18]. However, the molecular mechanisms underlying TGM3 and cancers remain elusive, especially in cSCC.

Epidermal keratinization is a unique circumstance of programmed cell death and terminal differentiation of epidermal keratinocytes [19]. Keratinocytes in the basal layer of the epidermis undergo differentiation and proliferation to populate the upper differentiated layers. However, malignant transformation of these cells can give rise to cSCC [20]. It has been shown that the expression of late differentiation markers, such as involucrin, loricrin, Keratin (KRT) 10, and small proline-rich proteins, is deregulated in skin samples with keratinocyte hyperproliferation disorders, including psoriasis vulgaris, basal cell carcinoma, and squamous cell carcinoma [21, 22]. Therefore, understanding the role of markers involved in keratinocyte differentiation during the development of squamous cell carcinoma is crucial for comprehending the pathogenesis of cSCC.

In this study, we have identified Transglutaminase 3 (TGM3) as a novel cSCC tumor suppressor that regulates the PI3K-AKT axis. Loss of TGM3 results in activation of the PI3K-AKT pathway, which promotes cSCC cell proliferation, migration, invasion, and epithelial-mesenchymal transition (EMT). Deguelin effectively blocks TGM3 deficiency-induced PI3K-AKT activation and cSCC carcinogenesis. Furthermore, TGM3 serves as a novel cSCC tumor differentiation marker through binding to KRT14 and promoting its degradation.

## Material and methods

### Ethical approval of the study protocol

This study involving human participants (Approval No. XHEC-D-2022-116) and animal models (Approval No.XHEC-F-2022-008) were scrutinized and authorized by the ethics committee of Xinhua Hospital, Shanghai Jiaotong University School of Medicine. And informed consent was obtained from each patient before clinical data analyses.

### Patients and specimens

A total of 127 samples of human cSCC and the adjacent normal skin tissues were obtained from Dermatology Center of Xinhua Hospital (Shanghai, China) between March 2015 to June 2023. Half of each specimen was immediately frozen in liquid nitrogen until total protein or RNA was extracted, while the other half was embedded in paraffin for pathological diagnosis and immunohistochemical staining (IHC) (127 samples were used for IHC analyses). Among them, 12 tumor tissues and adjacent non-tumor tissues were randomly selected for western blotting and 72 tumor tissues and adjacent non-tumor tissues were used for real-time reverse transcription-quantitative polymerase chain reaction (RT-qPCR). The adjacent normal tissues were ≥2 cm away from tumor tissues.

### Inclusion and exclusion criteria

The inclusion criteria were (i) provision of comprehensive data for patients; (ii) basal cell carcinoma was excluded under histopathology; (iii) patients do not have cutaneous diseases; (iiii) Clinically and pathologically diagnosed patients with cSCC; (iiiii) The primary site of the tumor is limited to the head, neck and face. The exclusion criteria were (i) non-provision of comprehensive data for patients; (ii) patients were diagnosed with basal cell carcinoma; (iii) patients also suffer from other skin diseases; (iiii) Patients who have received radiotherapy, chemotherapy, immunotherapy, or other systemic or local treatments before surgical resection.

### Clinical samples of normal skin and cSCC in public databases

RNA-Seq data comprising 101 samples of adjacent normal tissue (ANT) and 79 samples of cutaneous squamous cell carcinoma (cSCC) were acquired from the GEO database. This dataset encompasses a total of seven distinct datasets, namely GSE2503, GSE32628, GSE39612, GSE42677, GSE45164, GSE45216, and GSE53462, which were utilized for the purpose of investigating TGM3 mRNA expression. The data underwent a standardization process prior to analysis.

### Cell culture

The cSCC lines (SCL-1, A431, HSC-1, and HSC-5) and a keratinocyte cell lines (HaCaT) were obtained from Institute of Dermatology, Shanghai Jiaotong University School of Medicine. All cells were free of mycoplasma. The A431, HaCaT, HSC-1 and HSC-5 cells were cultured in Dulbecco’s modified Eagle’s medium (Gibco, USA), while the SCL-1 cells were cultured in RPMI-1640 medium (Gibco, USA). All cell lines were supplied with 10% fetal bovine serum (Gibco, USA) and 1% penicillin-streptomycin. Finally, all the cell lines were incubated in a humidified incubator at 37 °C in an atmosphere of 5% CO_2_.

### Antibodies

All antibodies and dilutions used for western blot, immunohistochemistry, immunofluorescence, and immunoprecipitation were described in the Supporting Table 1.

### Cell transfection

The shRNAs of *TGM3* were designed for the target sequence: shA was CCTTGGCTCTAACGAAAGA, shB was TGAGTGCCATGATCAATAG, and shC was ACAAACCGAATTGGCATGA. Synthesized and purified TGM3 gene fragments were inserted into a lentivirus vector (Hanyin, China) termed TGM3- “overexpression” (OE) (NM_003245.4). A recombinant lentivirus was generated from 293T cells using Lipofectamine^®^ 3000 (Invitrogen, USA) and transfected into the cSCC cell lines SCL-1, HSC-1, A431 and HSC-5 were mediated by polybrene (Maokangbio, China). Cells were infected with this virus or the control virus, and then selected by puromycin for 7 days before use for other assays.

### Cell stimulation

For stimulation of HaCaT cells, 1.0 × 10^5^ cells/well in 2 mL were seeded in a six-well plate and cultured in a medium with a calcium concentration of 1.8 mM for 10 days. The medium was changed every 2 days. After 5 days or 10 days, the medium was removed, and cells were lysed for western blotting. Meanwhile, HaCaT cells were seeded in 3.5-mm culture plates at 5 × 10^4^ cells/well and cultured in a medium with a calcium concentration of 1.8 mM for 10 days, and the medium was changed every day. Cellular RNA was extracted for RT-qPCR from day-0 to day-10.

### Western blotting

The total protein in tissues and cells was lysed using RIPA lysate buffer with fluoride (Beyotime Institute of Biotechnology, China). The protein concentration was then quantified with bicinchoninic acid Kit (Beyotime Institute of Biotechnology, China). To isolate the protein, sodium dodecyl sulfate-polyacrylamide gel electrophoresis (SDS-PAGE) was performed, and the protein was then transferred to polyvinylidene difluoride (PVDF) membranes (Millipore, USA). After being blocked with 5% bovine serum albumin (BSA) solution, the PVDF membranes were sequentially incubated with primary antibodies and horseradish peroxidase-conjugated secondary antibodies.

### Immunohistochemistry

Paraffin-embedded specimens were placed in a heated incubator for 20 min. The paraffin slices were immersed successively in xylene to remove the paraffin, followed by absolute ethanol and a 90% ethanol-water mixture to dehydrate them. They were then treated with 3% H_2_O_2_ for 15 min to inactivate the endogenous peroxidase. Next, the sections were treated with normal serum blocking solution at room temperature for 1 h, followed by incubation with indicated antibodies at 4 °C overnight. The following day, the sections were incubated with the indicated secondary antibodies. Positive staining was visualized with DAB (3, 3-diaminobenzidine) (Thermo Fisher Scientific, USA), and then counterstained with hematoxylin.

### RNA extraction and real-time PCR

Total RNA was extracted from tissues and cells to generate single-stranded complementary DNA using TRIzol® Reagent (Invitrogen, USA) and EZ-press RNA Purification kit (EZ Bioscience, China) according to manufacturer protocols. Subsequently, qPCR was performed using the SYBR Green qPCR Master Mix (EZ Bioscience, China) on a QuantStudio™ 3 real-time PCR system (Applied Biosystems, USA). The thermocycling conditions were as follows: hot-start DNA polymerase activation (95 °C for 5 min), 40 cycles (95 °C for 15 s and 60 °C for 30 s), and melt-curve analyses (95 °C for 15 s, 60 °C for 60 s, 95 °C for 30 s, and 60 °C for 15 s). The following primer pairs (forward and reverse, respectively) were used for qPCR: 5′-TGGCATGATTGGCTGGAACT-3′ and 5′-CCAGCAAGCACACCATTGTC-3′ for TGM3; 5′-AAGGTGACAGCAGTCGGTT-3′ and 5’-AACTGGTGTCGTGGAGTC-3’for β-actin.

### Evaluation of biological function of PI3K-AKT and EMT-related gene expressions

Transcript-level quantification, analysis of differentially expressed transcript (DEGs), cluster analysis, GO and KEGG enrichment. Hierarchical cluster analysis of DEGs was performed to explore transcripts expression pattern. GO enrichment and KEGG pathway enrichment analysis of DEGs were respectively performed using R based on the hypergeometric distribution.

### Co-Immunoprecipitation (Co-IP)

HaCaT cells were dissolved with IP lysis buffer (Applygen Technologies, China) containing an EDTA-free InStab™ Protease Inhibitor Cocktail (Yeasen Biotechnology, China). Then, cell lysates were incubated with protein A/G beads (Santa Cruz Biotechnology, USA) and indicated antibodies overnight at 4 °C on a rotator, and then washed thrice in IP wash buffer. Next, 20 μl of loading buffer (Yeasen Biotechnology, China) was added to the pellet, followed by boiling for 10 min and analysis using western blotting.

### Cell Counting Kit-8 (CCK-8) assay

Cell proliferation was assessed using the CCK-8 assay (Takara, Japan) according to manufacturer’s instructions. In brief, transfected cells were seeded into 96-well plates (5 × 10^3^ cells/well) and cultured for 24, 48, 72, 96, or 120 h. After incubation, CCK-8 reagent was added to each well for 2 h. Then the optical density was measured at 450 nm using a microplate reader (Bio-Rad Laboratories, USA).

### Colony-formation assay

Cells were seeded into 6-well plates (200 cells/well) for colony-formation assays, and the medium was replaced every 2 days. After 2 weeks, the colonies were fixed with 10% formaldehyde and stained with 5% Giemsa staining solution (Beyotime Institute of Biotechnology, China). The average number of colonies was then counted under a light microscope and photographed using a camera (EOS850D, Canon, Japan).

### Transwell assay

Transwell assay, as previously described [23], was performed using chambers with 8μm pore size (Corning, USA). For the cell migration assay, 5.0 × 10^4^ cells were seeded into the upper chamber in serum-free medium, while RPMI-1640 medium containing 12% fetal bovine serum was placed in the lower chamber. After a 24-h incubation, the cells remaining in the upper chamber were removed, while the cells that had migrated to the lower chamber were fixed with 4% paraformaldehyde and stained with crystal violet. For the cell invasion assay, each chamber was pre-coated with 50 μl BD Matrigel mixture (diluted at 1: 5 with serum-free DMEM) and incubated at 37 °C for 1 h. After 24-h incubation, the cells in the upper chamber were wiped off, and the cells that had invaded the lower chamber were stained with 0.1% crystal violet. The cells from three randomly selected fields were then photographed using an IXplore inverted microscope (Olympus, Japan).

### Xenograft model

Female NOD/SCID mice, aged 4 weeks and weighing between 16 and 18 grams, were procured from GemPharmatech Co., Ltd. and maintained under specific pathogen-free conditions. For each group, A431 cells, sh-normal control (NC) A431 cells, TGM3-shA A431 cells, and TGM3-shC A431 cells were injected subcutaneously into the left armpit of NOD/SCID mice (1 × 10^7^ cells in 500 μL of PBS). Once the tumor xenografts became apparent, the minimum width (W) and maximum length (L) of tumor size were measured every 6 days using a vernier caliper. Tumor volumes (V) were calculated using the formula V = LW^2^/2. Mice were sacrificed 50 days after tumor injection, and tumor weights were determined. The tumors were stored in liquid nitrogen or embedded in paraffin for hematoxylin-eosin (H&E) and IHC staining.

### Statistical analyses

GraphPad Prism Version 8.0. (GraphPad, USA) was used to analyze data. All data are presented as the mean ± standard deviation. The Shapiro-Wilk test was performed to assess the data distribution. For comparisons between two groups, an unpaired two-tailed *t*-test or Mann–Whitney two-tailed test was used. For comparisons among multiple groups, ordinary one-way ANOVA with Dunnett’s post hoc or Kruskal–Wallis test followed by Dunn’s post hoc, depending on whether the data followed a Gaussian or non-Gaussian distribution, was used. *P* < 0.05 was considered statistically significant.

## Results

### Loss of TGM3 correlates with poor differentiation and a high recurrence rate of cSCC

**Fig. 1:**
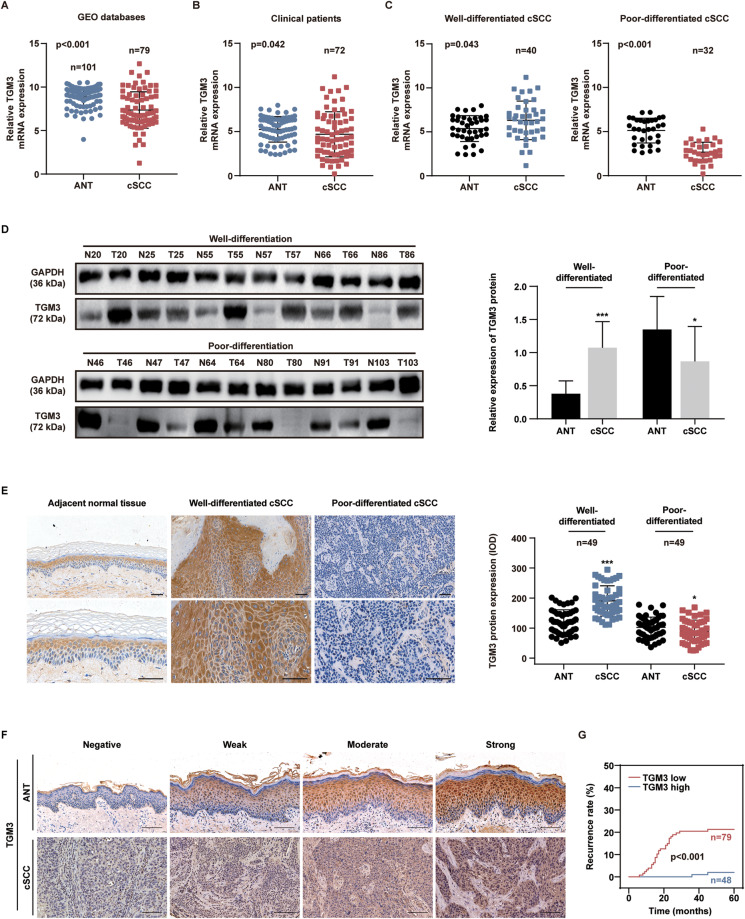
Loss of TGM3 correlates with poor differentiation and prognosis of cSCC. **A** The relative TGM3 mRNA expression level in 101 cSCC samples and 79 normal samples from GEO cancer database. **B** The relative TGM3 mRNA level in 72 paired cSCC and adjacent non-tumor issues (ANT). **C** The relative TGM3 mRNA level in paired cSCC and adjacent non-tumor issues (ANT). Left panel: The relative TGM3 mRNA level in 40 paired well-differentiated cSCC and adjacent non-tumor issues (ANT). Right panel: The relative TGM3 mRNA level in 32 paired poor-differentiated cSCC tumors and adjacent non-tumor issues (ANT). **D** TGM3 protein expression was higher in well-differentiated cSCC tumors, while it was lower in poor-differentiated cSCC tumors compared to corresponding adjacent normal tissues. n = 12. **E** The immunohistochemistry (IHC) staining scores of TGM3 were determined in 49 pairs of well-differentiated cSCC and adjacent non-tumor tissues, as well as poor-differentiated cSCC and ANT specimens, respectively. Scale bar, 30 μm. **F** Representative IHC staining intensity of TGM3 in cSCC tumors and adjacent non-tumor tissue. Scale bar, 200 μm. **G** Low TGM3 expression is associated with significantly higher recurrence rate. n = 127. All experiments were repeated at least three times. Results were expressed as mean ± SD; n.s. not significant; *P < 0.05; **P < 0.01; ***P < 0.001; Mann-Whitney (**A**; **E** left panel); Wilcoxon (**B**; **C**, left panel; **D**, left panel) Paired two-tailed *t* test (**C**, right panel; **D**, right panel); Kruskal–Wallis one-way ANOVA (**E**); log-rank *t* test (**G**).

Consistent with the analysis of the cSCC cohort using the GEO database (Fig. 1A), we observed that the mRNA expression of *TGM3* was significantly lower in cSCC tumors compared to corresponding adjacent normal tissues (Fig. 1B). Notably, we observed that *TGM3* mRNA expression was higher in well-differentiated cSCC tumors compared to corresponding adjacent normal tissues, while it was lower and even absent in poor-differentiated cSCC tumors (Fig. 1C). Additionally, the findings were further supported through western blot analysis of randomly selected pairs of cSCC samples and IHC staining images. These results consistently demonstrated a significant upregulation of TGM3 protein expression in well-differentiated cSCC tissues and a downregulation of TGM3 expression in poor-differentiated cSCC tissues compared to adjacent normal tissues (Fig. 1D, E). To assess the clinical significance of TGM3 expression in cSCC, we quantified TGM3 expression and divided patients into high (moderate and strong) and low (negative and weak) TGM3 expression groups. We observed a negative correlation between TGM3 expression and significantly higher recurrence rates (Fig. 1F–G). Collectively, these results suggest that low TGM3 expression is associated with poor differentiation and a high recurrence rate of cSCC, and could serve as a potential biomarker for predicting poor differentiation in cSCC.

### TGM3 inhibits cSCC cell proliferation, migration and invasion in vitro, and suppresses tumor growth in vivo

**Fig. 2:**
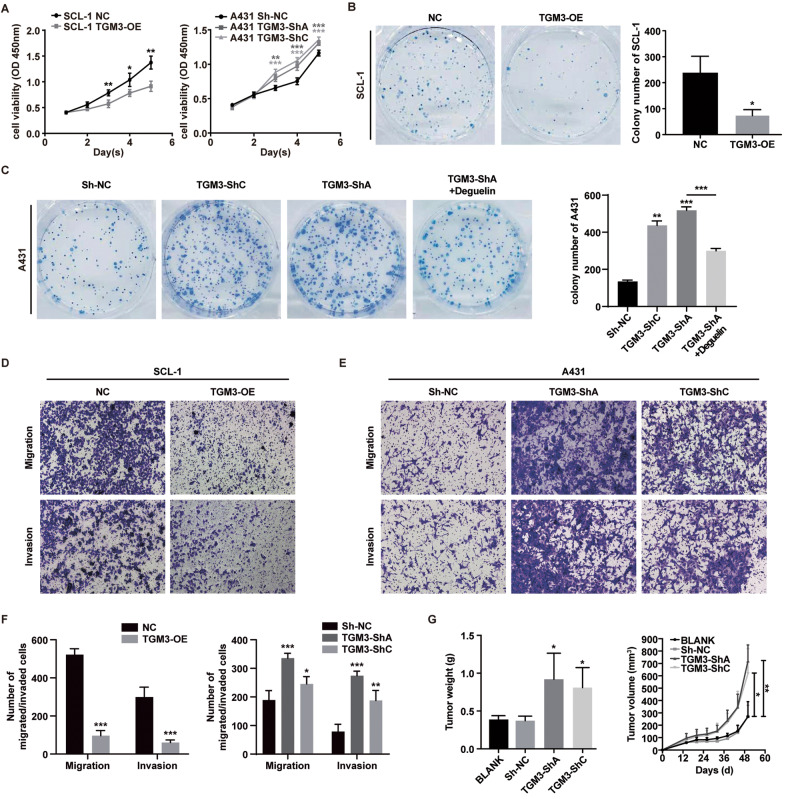
TGM3 inhibits cSCC cell proliferation, migration and invasion in vitro, and suppresses tumor growth in vivo. **A** Effects of TGM3 knockdown and overexpression on cSCC cell proliferation. CCK-8 assays were performed with the stable cells above. n = 5. **B** Effects of TGM3 overexpression on cSCC cell colony formation. Relative colony numbers were counted and shown in the right panel. n = 5. **C** Effects of TGM3 knockdown on cSCC cell colony formation. Relative colony numbers were counted and shown in the right panel. n = 5. **D** Effects of TGM3 overexpression on cSCC cell migration and invasion. **E** Effects of TGM3 knockdown on cSCC cell migration and invasion. **F** Relative cell numbers in SCL-1 (left panel) and A431 cells (right panel). n = 5; Scale bar, 100 μm. **G** Quantification of tumor weight (left panel) and tumor volume (right panel) of subcutaneous A431 xenograft tumor for each group. n = 5. All experiments were repeated three or five times. Results were expressed as mean ± SD; n.s. not significant; *P < 0.05; **P < 0.01; ***P < 0.001; Two way ANOVA Sidak test (**A** left panel); Two way ANOVA Dunnett test (**A** right panel; **G** right panel); Unpaired two-tailed *t* test (**B**; **C**; **F**, left panel; 2G, left panel); Welch’s test (**F**, left panel; **G**, left panel); Brown-Forsythe and Welch ANOVA tests (**C**; **F**, right panel); Mann-Whitney (2 F, right panel).

We assessed the endogenous expression of TGM3 in keratinocytes and cSCC cell lines. Compared to primary human keratinocytes and the normal keratinocytes cell line HaCaT, TGM3 expression was significantly reduced in cSCC cell lines, particularly in HSC-1 and SCL-1 (Fig. S1A). To investigate the function of TGM3 in cSCC, we established stable TGM3 knockdown A431 and HSC-5 cell lines, as well as TGM3 overexpressing SCL-1 and HSC-1 cell lines (Fig. S1B, C). Our findings demonstrate that TGM3 overexpression suppressed cSCC cell proliferation, whereas TGM3 knockdown enhanced cell proliferation (Fig. 2A–C). Cell migration ability revealed a significant increase in TGM3 knockdown A431 cells and a decrease in TGM3 overexpression SCL-1 cells. Similarly, transwell invasion assays indicated that overexpression of TGM3 significantly decreased cell invasion ability, while TGM3 knockdown increased cell invasion in cSCC cells (Fig. 2D–F).

To assess the impact of TGM3 on tumor growth in vivo, we established a subcutaneous xenograft tumor model in NOD/SCID mice. The results revealed a significant increase in tumor growth rate, tumor weight, and tumor volume in mice injected with TGM3-knockdown A431 cells compared to control A431 cells (Figs. 2G, S3A, B). Collectively, our findings suggest that TGM3 acts as a tumor suppressor in cSCC both in vitro and in vivo.

### TGM3 promoted epidermal differentiation through enhancing Keratin 14 degradation in cSCC

**Fig. 3:**
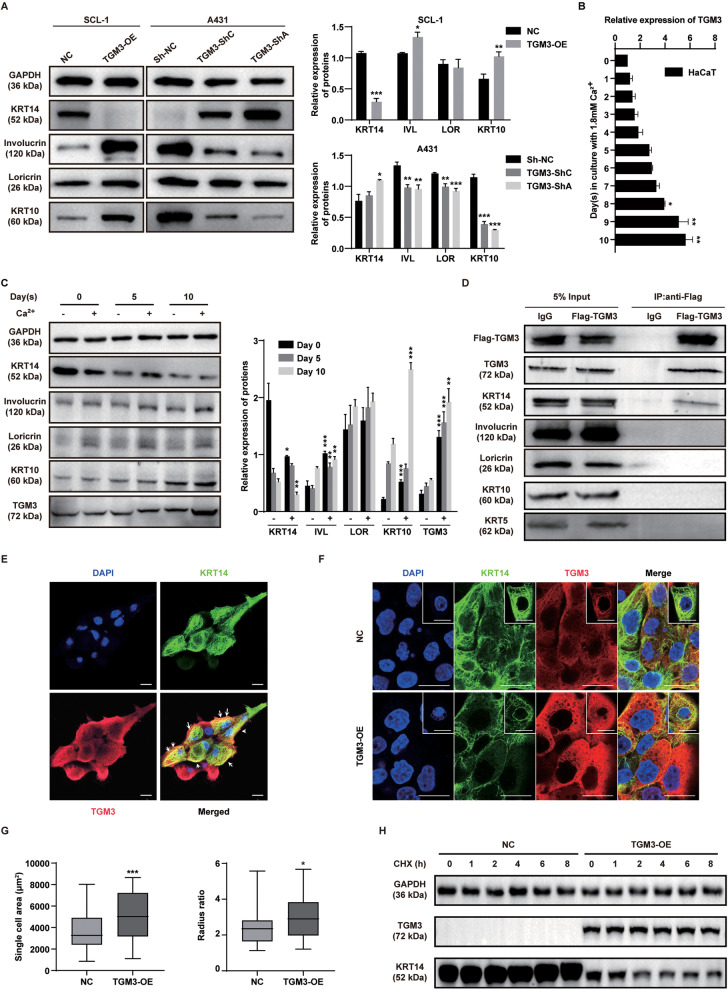
TGM3 promoted epidermal differentiation through enhancing Keratin 14 degradation in cSCC cells. **A** The effects of TGM3 overexpression and knockdown on the markers of keratinization differentiation were evaluated in SCL-1 and A431 cells. The quantification of each immunoblotting result was displayed in the right panels. n = 5. **B** TGM3 mRNA expression in the extracellular calcium induced HaCaT cell differentiation model. n = 5. **C** Differences in the expression of keratotic differentiation markers in models of HaCaT cell differentiation with and without extracellular calcium. Quantification of the immunoblotting results were shown in right panel. n = 5. **D** Co-Immunoprecipitation test showed TGM3 interacts specifically with KRT14, rather than KRT5, KRT10, involucrin, or loricrin in A431 cells. **E** Immunofluorescence showed co-localization (indicated by white arrow) of TGM3 and KRT14 in A431 cells. Scale bar, 25 μm. **F** Immunofluorescence analysis demonstrated that TGM3 overexpression led to the degradation of KRT14 in SCL-1 cells. Cell form of TGM3- overexpression SCL-1 cells results in a reverse EMT-like morphological transition, transforming from irregular polygonal fibroblast-like shapes to dense cobblestone-like epithelial structures. Scale bar, 25 μm. **G** The morphological changes of TGM3-overexpression SCL-1 cells compared with control cells. n = 44 **H** Hydroxychloroquine (CHX) stability assays revealed that TGM3 overexpression TGM3 suppressed the degradation of KRT14. All experiments were repeated at least three times. Results were expressed as mean ± SD; n.s. not significant; *P < 0.05; **P < 0.01; ***P < 0.001; Two-way ANOVA Sidak test (A, upper right panel); Unpaired two-tailed *t* test (**A**, lower right panel; **C**); Kruskal–Wallis one-way ANOVA (**B**); Brown–Forsythe and Welch ANOVA tests (**C**); Sidak two-way ANOVA test (**C**); Welch’s test (**C**; **G**, left panel); Mann–Whitney (G, right panel).

Epidermal keratinization is a unique process of programmed cell death and terminal differentiation of epidermal keratinocytes. To examine the impact of TGM3 on keratinocyte differentiation in cSCC, we evaluated the expression of keratinization differentiation markers in TGM3-knockdown A431 cells and TGM3-overexpression SCL-1 cells. Knockdown of TGM3 in A431 resulted in the suppression of multiple markers of late keratinization differentiation, including KRT10, involucrin, and loricrin, while augmenting the expression of early keratinization differentiation markers KRT14. In contrast, markers of late keratinization differentiation proteins KRT10 and involucrin were up-regulated in TGM3-overexpressing SCL-1 cells, while KRT14 was significantly down-regulated. (Fig. 3A).

HaCaT cells were exposed to 1.8 mM extracellular calcium to promote cell differentiation. Ca^2+^ plays a pivotal role in cell differentiation and maturation, leading to a consistent upregulation of late keratinization markers KRT10 and involucrin over time. Simultaneously, TGM3 demonstrates a notable ascending trend during this process. Conversely, the expression of KRT14 progressively diminishes as the cells differentiate and mature. (Fig. 3B, C). Importantly, further mechanistic investigations revealed that TGM3 interacts specifically with KRT14, rather than KRT5, KRT10, involucrin, or loricrin in A431 cells (Fig. 3D). Immunofluorescence analysis confirmed the co-localization of TGM3 and KRT14 within A431 cells (Fig. 3E). Additionally, immunofluorescence analysis demonstrated that TGM3 overexpression led to the degradation of KRT14 in SCL-1 cells (Fig. 3F). The overexpression of TGM3 in SCL-1 cells results in a reverse EMT-like morphological transition, transforming from irregular polygonal fibroblast-like shapes to dense cobblestone-like epithelial structures. (Fig. 3F–G). After validating the interaction between TGM3 and KRT14, we then explored the underlying mechanism of TGM3-induced down-regulation of KRT14. Through employment of hydroxychloroquine (CHX) stability assays, we discovered that overexpression of TGM3 suppressed KRT14 degradation (Fig. 3H). These results suggest that TGM3 interacts with KRT14 to decrease its stability, thus contributing to the deregulation of epidermal differentiation in cSCC cells.

### Loss of TGM3 activates PI3K-AKT signaling pathway and triggers epithelial–mesenchymal transition (EMT) in cSCC

**Fig. 4:**
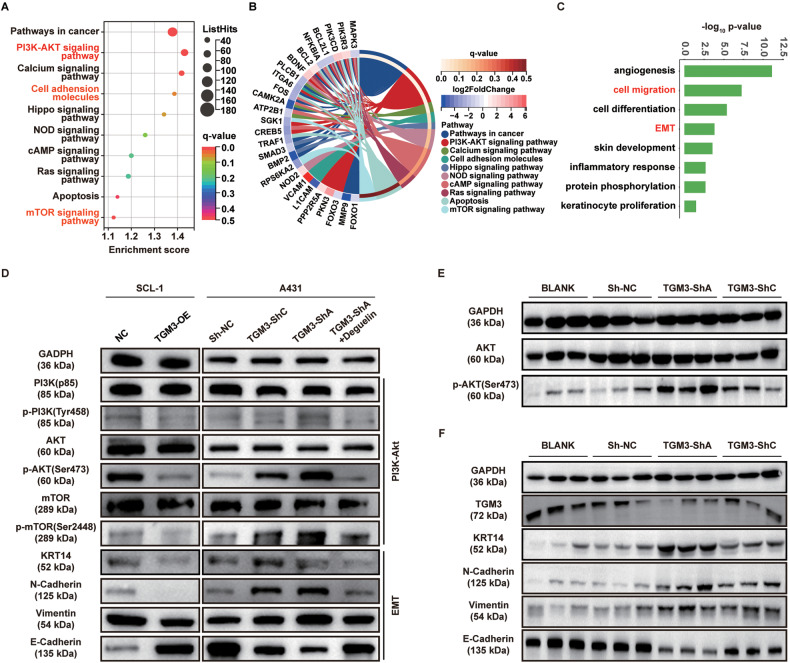
TGM3 inhibits EMT progression via PI3K-AKT signaling in cSCC cells. **A**, **B** KEGG pathways analysis of differentially expressed genes (DEGs) revealed a notable increase in the activity of the PI3K-AKT signaling pathway and Epithelial-mesenchymal transition (EMT) progression in TGM3-knockdown A431 cells. **C** GO pathway enrichment analysis of DEPs revealed marked up-regulation of key molecules involved in EMT progression. **D** Effects of TGM3 knockdown and overexpression on PI3K-AKT signaling and KRT14 and EMT markers in SCL-1 and A431 cells. **E** Effect of TGM3 knockdown on PI3K-AKT signaling in subcutaneous A431 xenograft tumor model. **F** Effect of TGM3 knockdown on the expression of KRT14 and EMT markers in subcutaneous A431 xenograft tumor model. All experiments were repeated at least three times.

To investigate the molecular mechanisms underlying the tumor-suppressing effect of TGM3, we performed RNA-sequence analysis on control and TGM3-knockdown A431 cells. Analysis of differentially expressed genes (DEGs) revealed that enriched pathways mainly included PI3K-AKT signaling, and EMT (Fig. 4A–C).

PI3K-AKT signaling pathway is a classic signaling pathway that plays a crucial role in carcinogenesis. Here, we observed a significant decrease in the expression of key molecules in the PI3K-AKT signaling pathway, including the PI3K(p85) Tyr458 phosphorylation, AKT Ser473 phosphorylation, and mTOR Ser2448 phosphorylation was significantly decreased in TGM3-overexpression SCL-1 and HSC-1 cells (Fig. 4D, upper left panel; Fig. S2A, C, left panel). Conversely, PI3K-AKT signaling was enhanced in TGM3-knockdown A431 and HSC-5 cells (Fig. 4D, upper right panel; Fig. S2B, C, right panel). Furthermore, TGM3 deficiency activates PI3K-AKT signaling pathway in subcutaneous A431 xenograft tumor model (Figs. 4E, S3C). Overall, these fundings suggest that TGM3 antagonizes PI3K-AKT signaling in cSCC.

EMT plays a crucial and intricate role in promoting tumor invasion and metastasis in epithelium-derived carcinomas [24]. The classic EMT phenotypes, including the downregulation of E-cadherin and the upregulation of N-cadherin, were observed in both TGM3-knockdown cSCC cells and the TGM3-knockdown subcutaneous xenograft tumor model (Fig. 4D, lower right panel; Figs. 4F; S2B, C, right panel, S2D, right panel, and S3D). Conversely, overexpression of TGM3 in cSCC cells inhibited EMT phenotypes (Fig. 4D, lower left panel; Fig. S2A, C, left panel and S2D, left penal). KRT14, which interacts with TGM3, serves as a biomarker for early differentiation of the skin and is considered a key molecule in the EMT process. Loss of TGM3 significantly decreased expression of KRT14 in cSCC cells and subcutaneous xenograft tumor models (Figs. 4D, E, S2A–D).

### Deguelin, PI3K-AKT inhibitor, reverses cSCC tumor growth induced by TGM3 knockdown both in vivo and in vivo

**Fig. 5:**
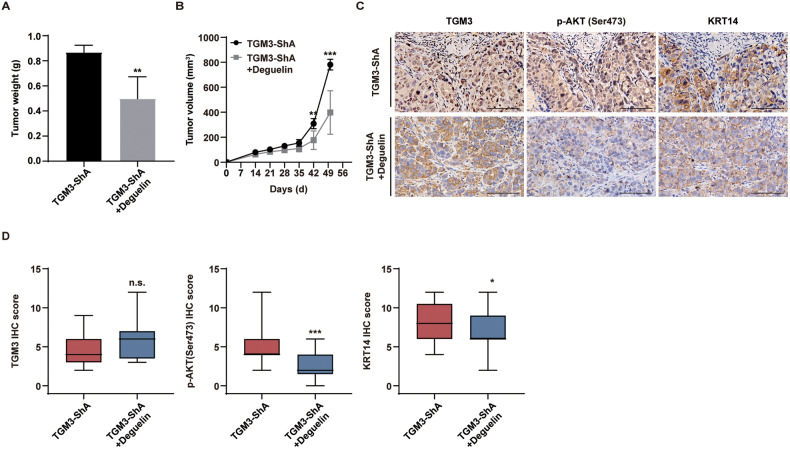
Deguelin, the PI3K-AKT inhibitor, reverses TGM3 knockdown-induced tumor growth cSCC in vivo*.* **A**, **B** Deguelin treatment reversed TGM3 knockdown-induced tumor growth in subcutaneous xenograft tumor model. **A**, **B** Quantification of tumor weight (A) and tumor volume (**B**) for each group. n = 5. **C** Representative IHC staining density of TGM3, AKT phosphorylation (Ser473) and KRT14 in subcutaneous tumor. Scale bar, 100 μm. **D** Deguelin reversed TGM3 knockdown-induced PI3K-AKT activation and EMT progression in subcutaneous xenograft tumor models. n = 5. Results were expressed as mean ± SD; n.s., not significant; *P < 0.05; **P < 0.01; ***P < 0.001; Welch’s test (**A**); Two-way ANOVA Sidak test (**B**); Mann–Whitney (**D**).

To further investigate the role of TGM3 knockdown-mediated PI3K-AKT signaling activation in cSCC development, we employed deguelin, a specific inhibitor of PI3K-AKT [25]. Our findings demonstrate that deguelin treatment reverses the abnormal cell proliferation induced by TGM3 knockdown in A431 cells (Fig. 2C). Deguelin therapy reversed the accelerated tumor growth, as manifested by tumor weight and tumor volume, caused by TGM3 knockdown in a subcutaneous xenograft tumor model (Figs. 5A, B, S3E). Additionally, deguelin therapy significantly reduced the expression of phosphorylated AKT in tumors derived from TGM3-knockdown cells (Fig. 5C, middle panel; 5D, middle panel). Deguelin also reversed TGM3 knockdown-induced KRT14 higher expression in xenograft tumor models. (Fig. 5C, right panel; 5D, right panel).

**Fig. 6:**
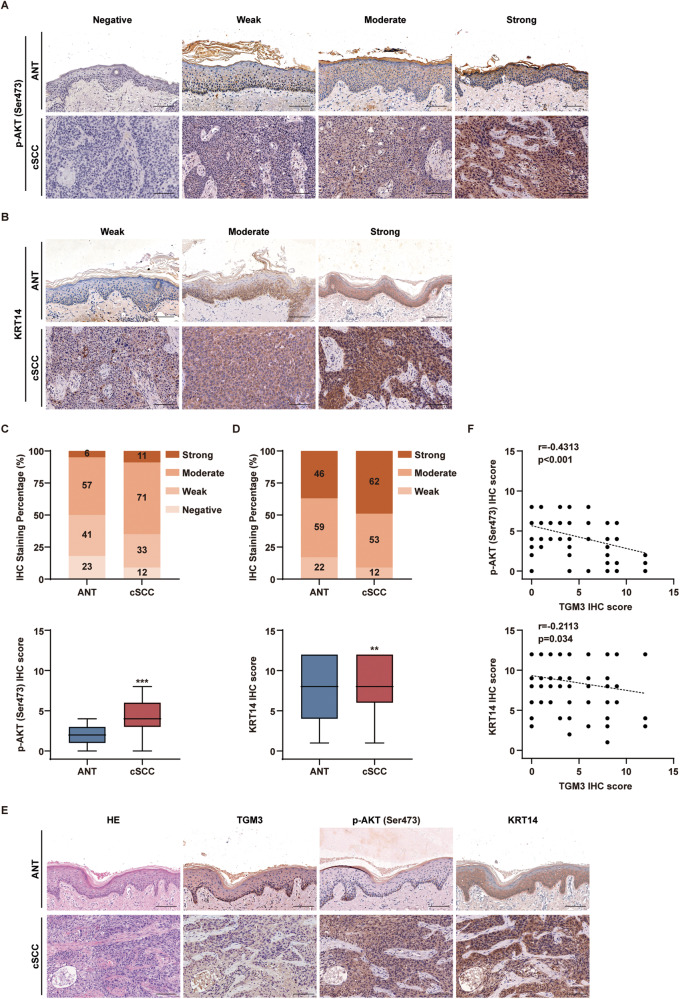
Loss of TGM3 associates with increased AKT activity and accelerated KRT14 in human cSCC tissues. **A** Representative IHC staining intensity of AKT phosphorylation (Ser473) in cSCC and adjacent non-tumor tissue. Scale bar, 200 μm. **B** Representative IHC staining intensity of KRT14 in cSCC and adjacent non-tumor tissue. Scale bar, 200 μm. **C** Scores of AKT phosphorylation (Ser473) IHC staining in cSCC and adjacent non-tumor tissue. n = 5. **D** Scores of KRT IHC staining in cSCC and adjacent non-tumor tissue. n = 5. **E** Representative images of H&E staining and IHC staining. The IHC staining of cSCC and adjacent non-tumor tissue demonstrated that TGM3 displayed an opposing pattern to AKT phosphorylation (Ser473) and KRT14.Scale bar, 200 μm. **F** The negative correlations between TGM3 protein level and AKT phosphorylation (Ser473) and KRT14 protein level in human cSCC and adjacent non-tumor tissue. n = 48. All experiments were repeated at least three times. Results were expressed as mean ± SD; n.s. not significant; **P < 0.01; ***P < 0.001; Welch’s test (**C**; **D**); Pearson correlation analysis (**F**).

To examine the correlation between TGM3 deficiency and abnormal activation of PI3K-AKT in human cSCC, we assessed the expression levels of TGM3, AKT Ser473 phosphorylation, and KRT14 in human cSCC samples. Our findings demonstrate that the expression of p-AKT (Ser473) and KRT14 was increased, while the expression of TGM3 was decreased in cSCC (Figs. 1A–E, 6A–D). A weak to moderate but significant negative correlation was observed between TGM3 expression and both p-AKT (Ser473) and KRT14 levels (Fig. 6E, F). Collectively, these data provide evidence for the association between TGM3 loss and PI3K-AKT activation in human cSCC.

## Discussion

In this study, we discovered a novel cSCC tumor suppressor gene, TGM3, which regulates the PI3K-AKT signaling pathway and the EMT process. Loss of TGM3 expression is observed in patients with poor-differentiated cSCC, and this is associated with higher recurrence rates. TGM3 inhibits the carcinogenesis of cSCC by regulating cell proliferation, migration, and invasion both in vitro and in vivo. Additionally, TGM3 interacts with KRT14 to weaken its stability. TGM3 deficiency reduces the degradation of KRT14 and disrupts keratinocytes differentiation, ultimately leading to the development of cSCC. (the related mechanism is shown in Graphical Abstract).

TGM3 is mainly expressed in the upper basal layer of stratified squamous epithelium. TGM3 is a regulatory protein in many types of cancer and can be used as a carcinoma marker [10, 15, 17, 26]. Downregulation or upregulation of TGM3 protein expression in distinct types of epithelia (not merely the epidermis) is regarded to be essential for activating multiple carcinogenic pathways and EMT modulation to elicit cells dedifferentiation as well as increased survival, migration, and metastasis of cells [6]. However, the role of TGM3 in cSCC and the underlying molecular mechanism are not known. Here, the expression of TGM3 in cSCC is inversely correlated with the differentiation of cSCC. TGM3 protein is weakly expressed in normal skin epithelium and strongly expressed in well-differentiated cSCC, while its expression is reduced or even eliminated in poorly-differentiated cSCC. This implies that TGM3 may participate in the differentiation level of cSCC. These findings are consistent with previous observations of various molecules in head and neck squamous cell carcinoma (HNSCC), laryngeal squamous cell carcinoma (LSCC), and cervical squamous cell carcinoma (CSCC). However, none of the literature has explained the specific mechanism behind these observations [17, 27, 28]. Our findings suggest that decreased TGM3 expression is inversely linked to the malignancy or differentiation potential of cSCC, serving as a biomarker for differentiation and potentially assisting skin pathologists in assessing the degree of cSCC differentiation. A possible explanation for this novel finding is that during the transition from normal skin to invasive cSCC, cells capable of producing sufficient TGM3 in specific microenvironments are transformed into well-differentiated cSCC, while cells expressing only low levels of TGM3 during the invasive process maintain poor differentiation. However, further research is needed to validate this hypothesis. In this study, our results indicate that as cSCC progresses to a more aggressive phenotype, loss of TGM3 expression leads to reduced differentiation.

PI3K-AKT signaling play a crucial role in the signal transduction of a variety of cellular processes [29]. In the differentiation process of keratinocytes, it mainly regulates cell metabolism and growth, promoting the differentiation of keratinocytes and the enhancement of the epidermal barrier [30–36]. In the development of squamous cell carcinoma, PI3K-AKT signaling promotes the proliferation and metastasis of tumor cells by regulating multiple metabolic pathways and cell cycle-related proteins [37–43]. Inhibition of PI3K-AKT signaling can effectively hinder the growth and spread of squamous cell carcinoma, providing guidance for the prevention and treatment of skin diseases [44–48]. In summary, the expression level and mechanism of PI3K-AKT in skin keratinocytes and squamous cell carcinoma exhibit both similarities and differences. A comprehensive understanding of the role of PI3K-AKT signal holds significant guiding value for the prevention and treatment of skin diseases. In our study, we found that TGM3 repress the proliferation, metastasis and EMT of cSCC cells via PI3K-AKT signaling pathway. EMT is a extensively studied process that involves the transformation of epithelial cells into mesenchymal cells, and PI3K-AKT is one of the most notable signaling pathways involved in EMT regulation [49–51]. Our findings demonstrated that loss of TGM3 enhanced the phosphorylation of PI3K, AKT, and mTOR. Deguelin, a PI3K-AKT inhibitor therapy reversed the accelerated tumor growth, as manifested by tumor weight and tumor volume, caused by TGM3 knockdown in a subcutaneous xenograft tumor model. Additionally, TGM3 deficiency resulted in increased expression of vimentin and N-cadherin, along with the loss of E-cadherin, which could be reversed through deguelin treatment. Taken together, our data provide strong evidence for the association between TGM3 loss and PI3K-AKT activation in human cSCC.

## Conclusion

Overall, our research has discovered a novel gene, TGM3, which mediates the PI3K-AKT signaling pathway through the EMT process, thereby promoting the development of cSCC. Additionally, we have found that TGM3 can serve as a biomarker for tumor differentiation to distinguish the malignant potential of tumors. This study not only discovered a feasible prognostic biomarker TGM3, but also revealed the potential for developing new treatment strategies for cSCC patients.

### Supplementary information

Supplementary figures and table

Full and uncropped western blots

Reproducibility checklist

## Data Availability

The datasets supporting the conclusions of this article are included within the article and its additional files.
