# Supplementary material for: Transglutaminase 3 regulates cutaneous squamous carcinoma differentiation and inhibits progression via PI3K-AKT signaling pathway-mediated Keratin 14 degradation
Source: Cell Death Dis. 2024 Apr 8;15(4):252. doi: 10.1038/s41419-024-06626-5 (PMC11001918; doi:10.1038/s41419-024-06626-5)
Supplement: Supplementary file 1 — Supplementary figures and table [file 41419_2024_6626_MOESM1_ESM.docx]

**Supplementary figures and table**

**
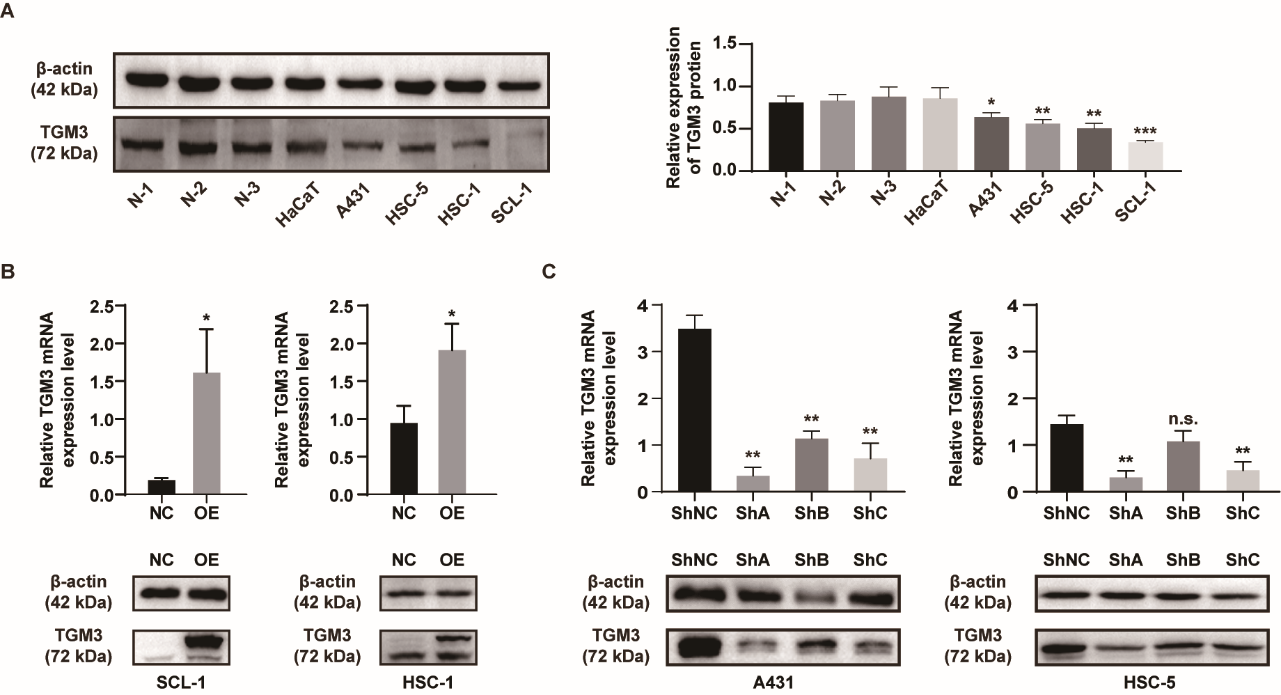
**

**Supplementary Figure S1. Establishment of stable TGM3-overexpression and TGM3-knockdown cSCC cell lines.**

**(A)** Relative TGM3 protein level in 3 human primary keratinocytes, normal keratinocytes cell line HaCaT and 4 cSCC cell lines. β-actin was used as loading control. **(B)** The efficiency of TGM3 overexpression in the SCL-1 and HSC-1 cell lines were detected by RT-qPCR and western blot. n = 5. **(C)** The efficiency of TGM3 knockdown in the A431 and HSC-5 cell lines were detected by RT-qPCR and western blot. n =5. All experiments were repeated at least three times. Results were expressed as mean ± SD; n.s., not significant; **P* < 0.05; ***P* < 0.01; ****P* < 0.001; Unpaired two-tailed t test (A; B, right panel); Welch‘s test (B, left panel); Brown-Forsythe and Welch ANOVA tests (C).

**
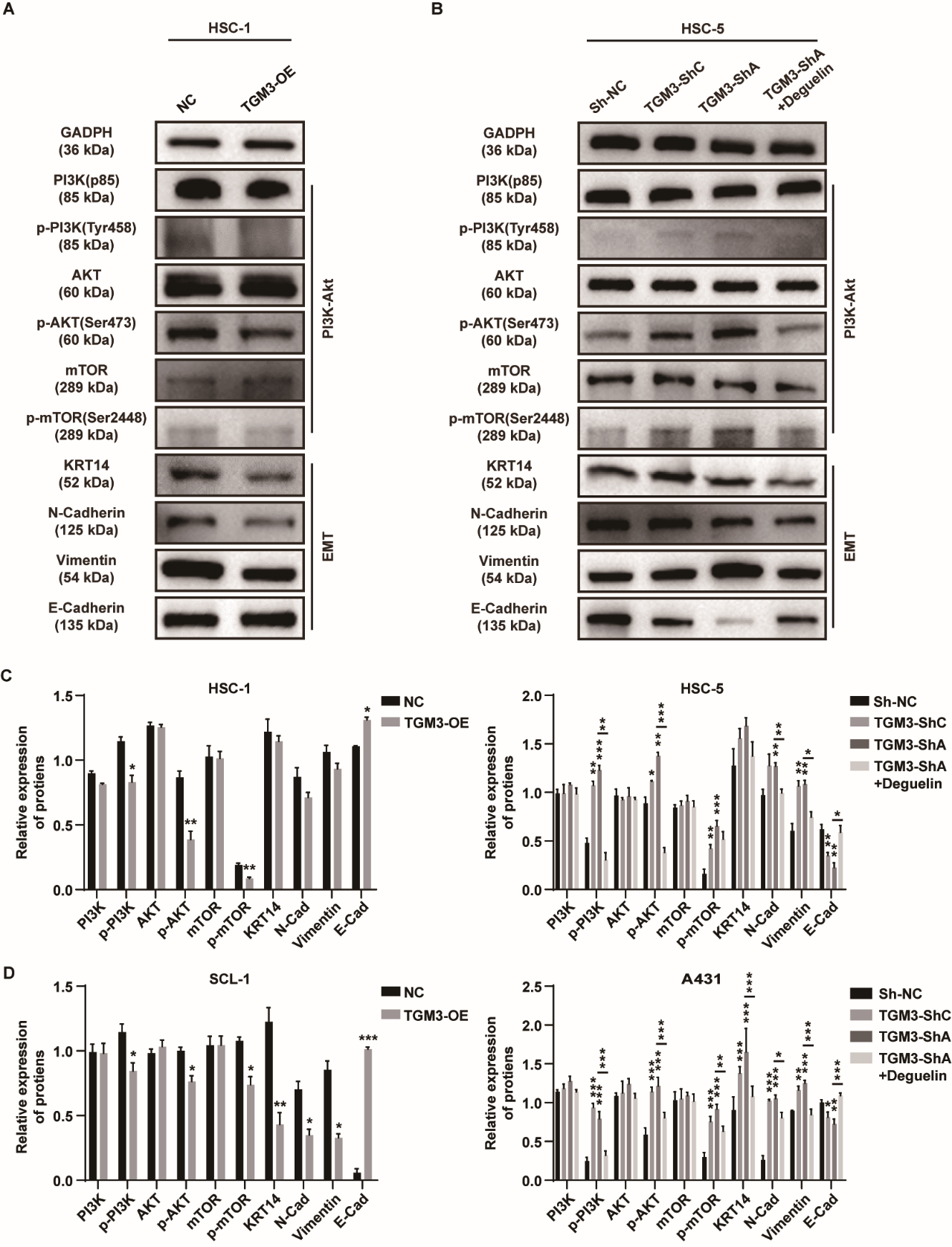
 Supplementary Figure S2. TGM3 inhibits EMT progression via PI3K-AKT signaling in cSCC cells.**

**(A)** Effects of TGM3 overexpression on PI3K-AKT signaling and EMT progression in HSC-1 cell line. **(B)** Effects of TGM3 knockdown on PI3K-AKT signaling and EMT progression in HSC-5 cell line. **(C)** Quantification of the immunoblotting results corresponding to panel A and B. n = 5. **(D)** Quantification of the immunoblotting results corresponding to panel Figure 4D n =5. All experiments were repeated at least three times. Results were expressed as mean ± SD; ns, not significant; **P* < 0.05; ***P* < 0.01; ****P* < 0.001; Two-way ANOVA Sidak test (C; D).


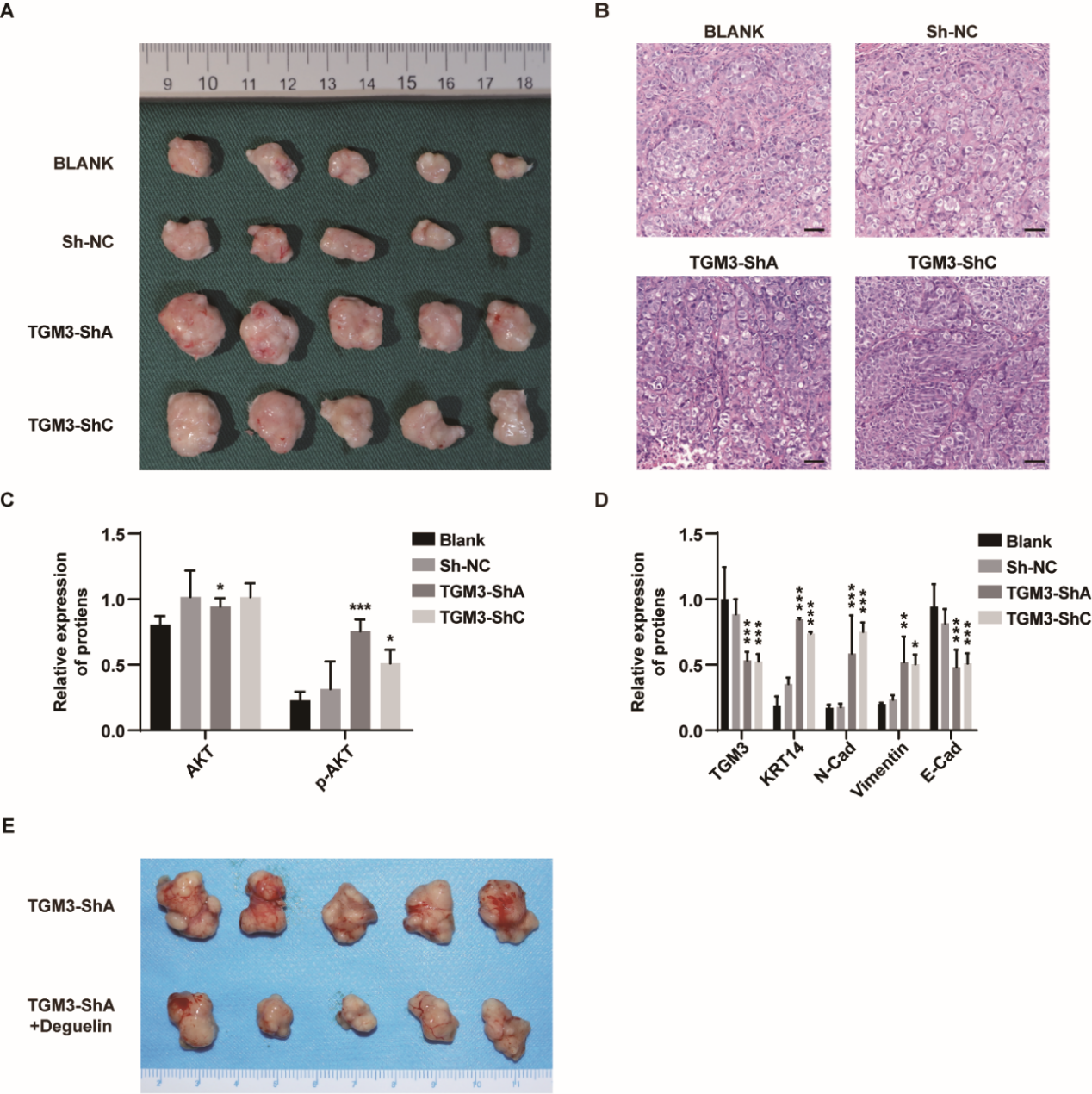


**Supplementary Figure S3. Deguelin, the PI3K-AKT inhibitor, reverses TGM3 knockdown-induced tumor growth cSCC *in vivo.***

**(A)** Effects of TGM3 knockdown on tumor growth in subcutaneous A431 human squamous cell carcinoma xenograft tumor model. n = 5. **(B)** Hematoxylin & Eosin (H&E) staining of tumor from subcutaneous A431 xenograft tumor model. Scale bar, 100 μm. **(C)** Quantification of the immunoblotting results corresponding to Figure 4E. n = 3. **(D)** Quantification of the immunoblotting results corresponding to Figure 4F. n = 3. **(E)** Macroscopic image of subcutaneous tumors derived from the mouse xenograft model demonstrates that Deguelin treatment reverses TGM3 knockdown-induced tumor growth in the A431 subcutaneous xenograft tumor model. All experiments were repeated at least three times. Results were expressed as mean ± SD; n.s., not significant; *P < 0.05; **P < 0.01; ***P < 0.001; Brown-Forsythe and Welch ANOVA tests (C); Two-way ANOVA Sidak test (D).

**Supplementary Table 1. Antibodies and dilutions used in this study.**

| Antibody | Dilutions for | | | | Company | Catalogue No. |
| --- | --- | --- | --- | --- | --- | --- |
|  | WB | IF | IP | IHC |  |  |
| TGM3 | 1:1000 | / | / | 1:100 | Atlas | HPA004728 |
|  | 1:1000 | 1:100 | / | / | Abclonal | A5856 |
| FLAG-Tag | 1:1000 | / | / | / | Cell Signaling Technology | 14793s |
|  | / | 1:100 | 1:200 | / | Sigma | F1804 |
| KRT14 | 1:2000 | / | / | / | Abcam | ab51054 |
|  | / | / | / | 1:200 | Proteintech | 10143-1-AP |
|  | / | 1:200 | / | / | Abcam | ab7800 |
| KRT5 | 1:2000 | / | / | / | Abcam | Ab52635 |
| KRT10 | 1:1000 | / | / | / | Abcam | ab76318 |
| Involucrin | 1:1000 | / | / | / | Abcam | ab181980 |
| Loricrin | 1:1000 | / | / | / | Abcam | ab176322 |
| AKT | 1:1000 | / | / | / | Cell Signaling Technology | 4691S |
| phosphorylated (p)-AKT  (Ser473) | 1:1000 | / | / | 1:100 | Cell Signaling Technology | 9271S |
| PI3K | 1:1000 | / | / | / | Cell Signaling Technology | 4257S |
| phosphorylated(p)-PI3K  (p85, Tyr458) | 1:1000 | / | / | / | Abcam | 4228S |
| mTOR | 1:1000 | / | / | / | Cell Signaling Technology | 2983S |
| phosphorylated (p)-mTOR  (Ser2448) | 1:1000 | / | / | / | Cell Signaling Technology | 5536S |
| E-cadherin | 1:1000 | / | / | / | Abcam | ab231303 |
| Vimentin | 1:1000 | / | / | / | Abcam | ab92547 |
| N-cadherin | 1:1000 | / | / | / | Abcam | ab76011 |
| GAPDH | 1:1000 | / | / | / | Beyotime | AF1186 |
| β-Actin | 1:1000 | / | / | / | Cell Signaling Technology | 4967 |
| HRP-linked Goat Anti Rabbit IgG | 1:5000 | / | / | / | Jackson ImmunoResearch | 115-035-003 |
| HRP-linked Goat Anti Mouse IgG | 1:5000 | / | / | / | Jackson ImmunoResearch | 111-545-003 |
| Alexa Fluor® 488 Goat Anti Mouse IgG | / | 1:500 | / | / | Abcam | Ab150105 |
| Alexa Fluor® 647 Goat Anti Rabbit IgG | / | 1:500 | / | / | Invitrogen | A21245 |

**Abbreviations:** WB, western-blot; IF, Immunofluorescence; IP, Immunoprecipitation; IHC, Immunohistochemistry.
