# Supplementary material for: Transglutaminase 3 regulates cutaneous squamous carcinoma differentiation and inhibits progression via PI3K-AKT signaling pathway-mediated Keratin 14 degradation
Source: Cell Death Dis. 2024 Apr 8;15(4):252. doi: 10.1038/s41419-024-06626-5 (PMC11001918; doi:10.1038/s41419-024-06626-5)
Supplement: Supplementary file 2 — Full and uncropped western blots [file 41419_2024_6626_MOESM2_ESM.pdf]

Original Western Blots

Figure 1D

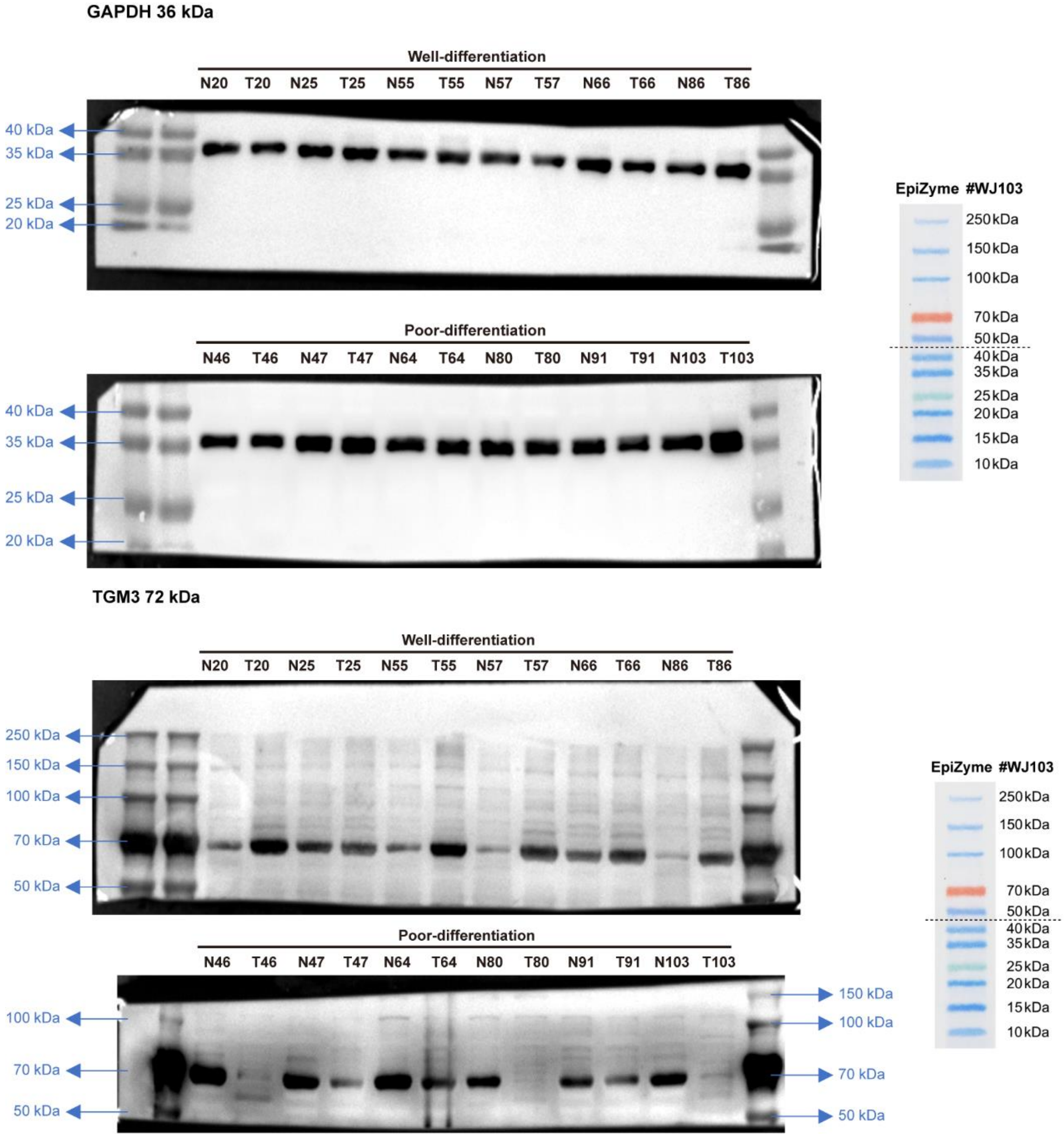

Figure 3A

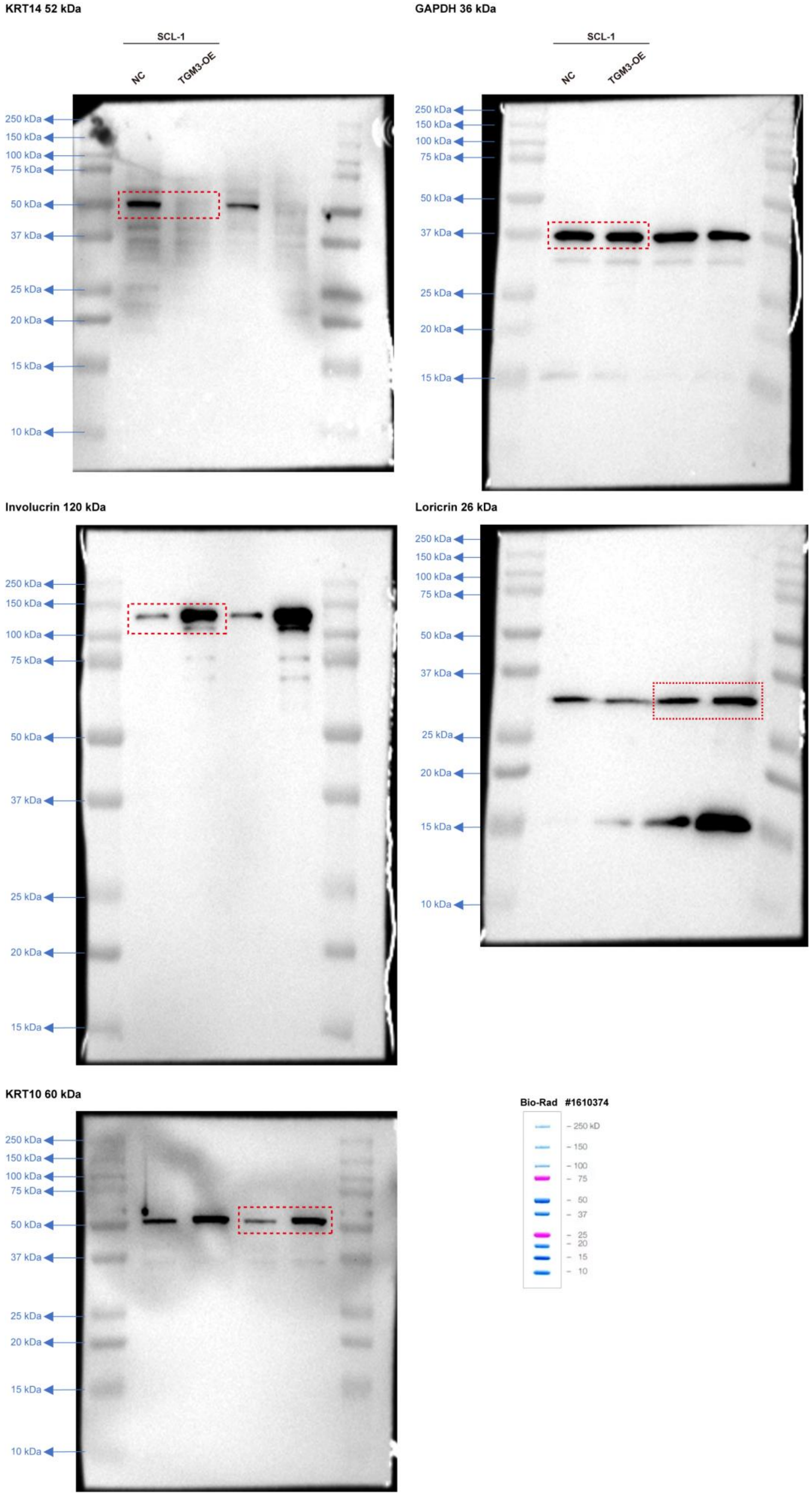

KRT14 52 kDa
SCL-1
NC
TGM3-OE
250 kDa
150 kDa
100 kDa
75 kDa
50 kDa
37 kDa
25 kDa
20 kDa
15 kDa
10 kDa
GAPDH 36 kDa
SCL-1
NC
TGM3-OE
Involucrin 120 kDa
Loricrin 26 kDa
KRT10 60 kDa
Bio-Rad #1610374
250 kD
150
100
75
50
37
25
20
15
10

KRT14 52 kDa

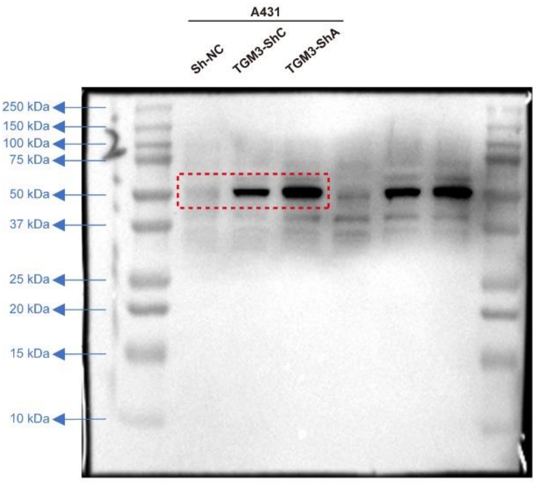

GAPDH 36 kDa

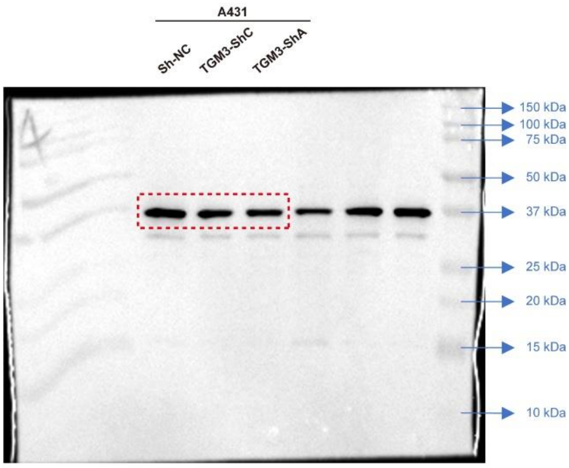

Involucrin 120 kDa

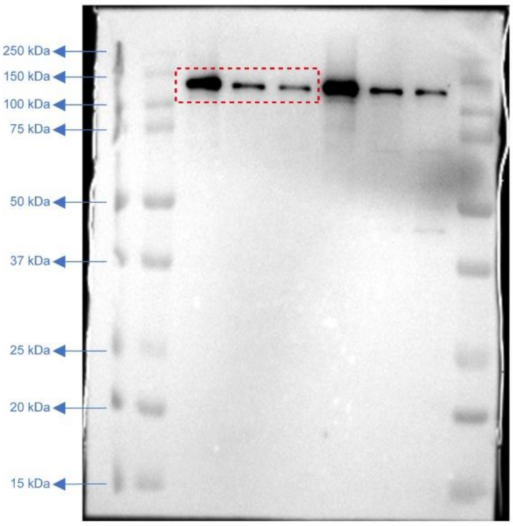

Loricrin 26 kDa

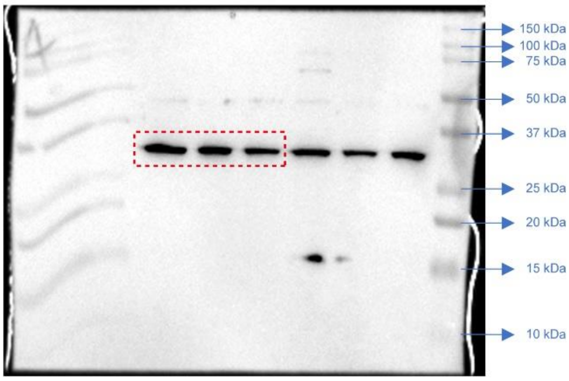

Bio-Rad #1610374

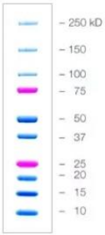

KRT10 60 kDa

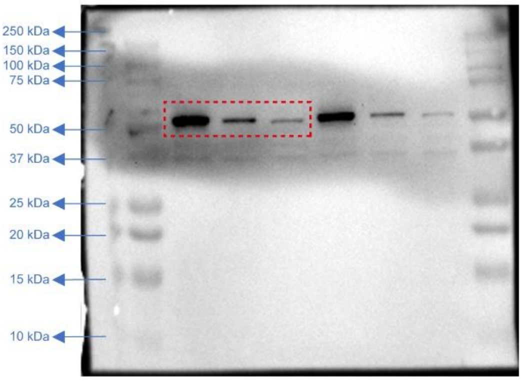

Figure 3D

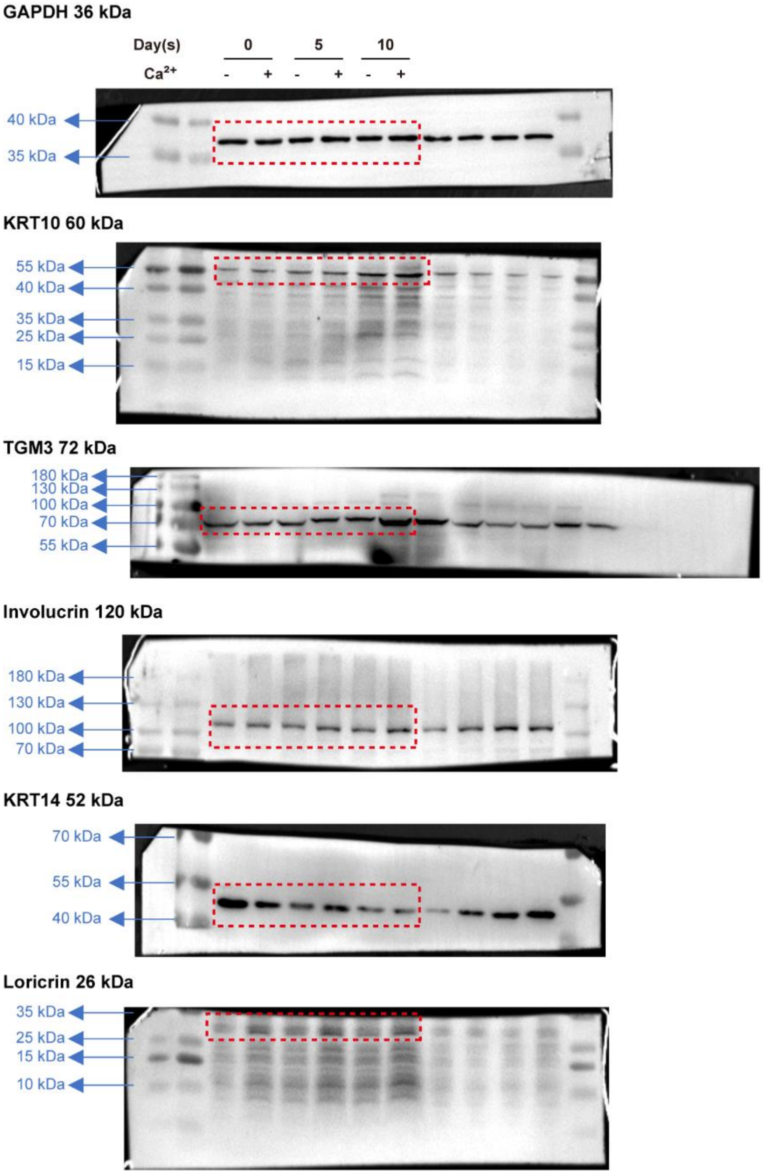
GAPDH 36 kDa
Day(s)
0
5
10
Ca2+
- + - + - +
40 kDa
35 kDa
KRT10 60 kDa
55 kDa
40 kDa
35 kDa
25 kDa
15 kDa
TGM3 72 kDa
180 kDa
130 kDa
100 kDa
70 kDa
55 kDa
Involucrin 120 kDa
180 kDa
130 kDa
100 kDa
70 kDa
KRT14 52 kDa
70 kDa
55 kDa
40 kDa
Loricrin 26 kDa
35 kDa
25 kDa
15 kDa
10 kDa

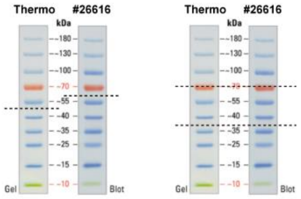
Thermo
#26616
kDa
180
130
100
70
55
40
35
25
15
10
Gel
Blot
Thermo
#26616
kDa
Gel
Blot

Figure 3E

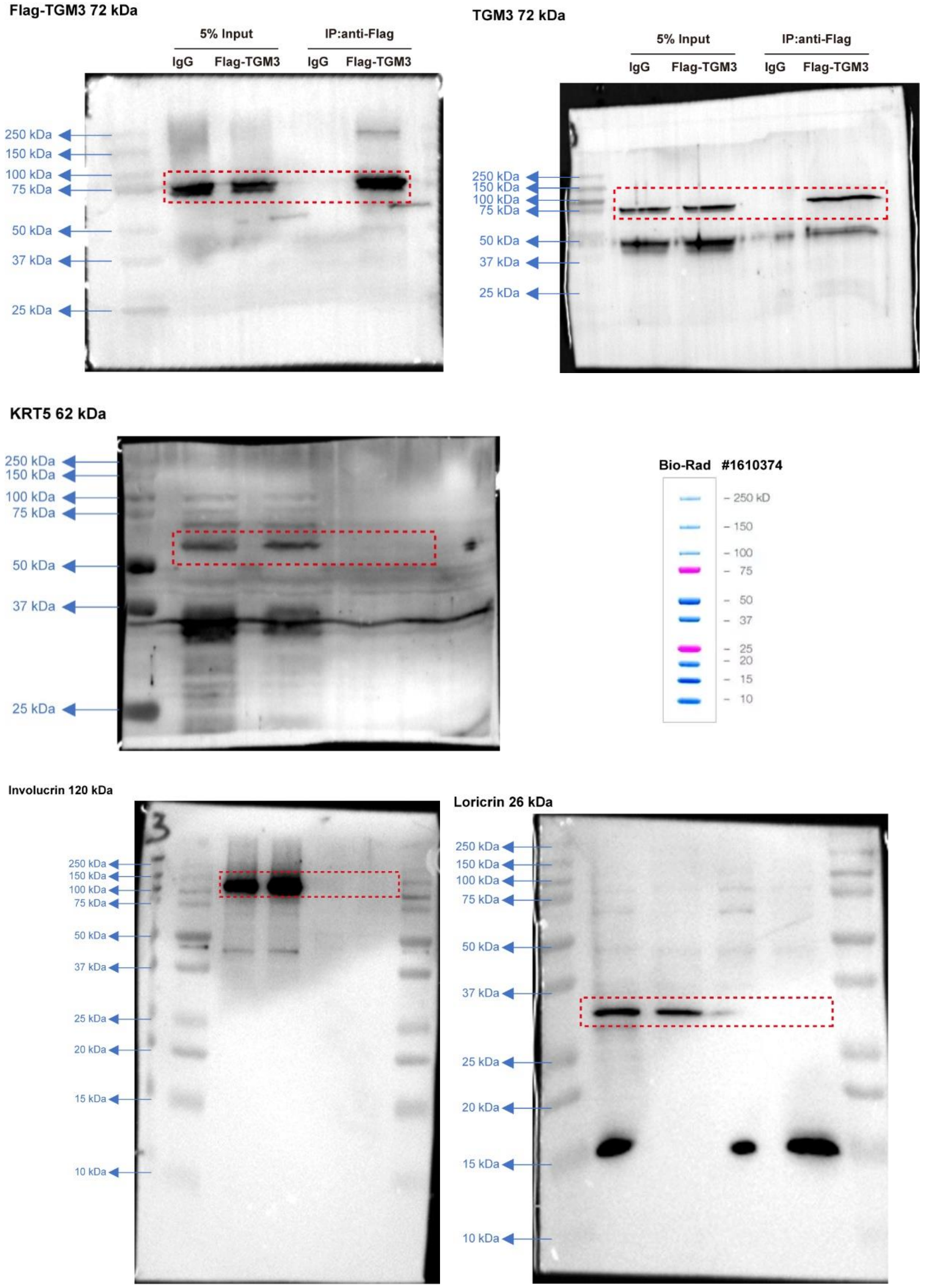

Flag-TGM3 72 kDa
5% Input
IP:anti-Flag
IgG
Flag-TGM3
IgG
Flag-TGM3
250 kDa
150 kDa
100 kDa
75 kDa
50 kDa
37 kDa
25 kDa
TGM3 72 kDa
5% Input
IP:anti-Flag
IgG
Flag-TGM3
IgG
Flag-TGM3
250 kDa
150 kDa
100 kDa
75 kDa
50 kDa
37 kDa
25 kDa
KRT5 62 kDa
250 kDa
150 kDa
100 kDa
75 kDa
50 kDa
37 kDa
25 kDa
Bio-Rad #1610374
250 kD
150
100
75
50
37
25
20
15
10
Involucrin 120 kDa
250 kDa
150 kDa
100 kDa
75 kDa
50 kDa
37 kDa
25 kDa
20 kDa
15 kDa
10 kDa
Loricrin 26 kDa
250 kDa
150 kDa
100 kDa
75 kDa
50 kDa
37 kDa
25 kDa
20 kDa
15 kDa
10 kDa

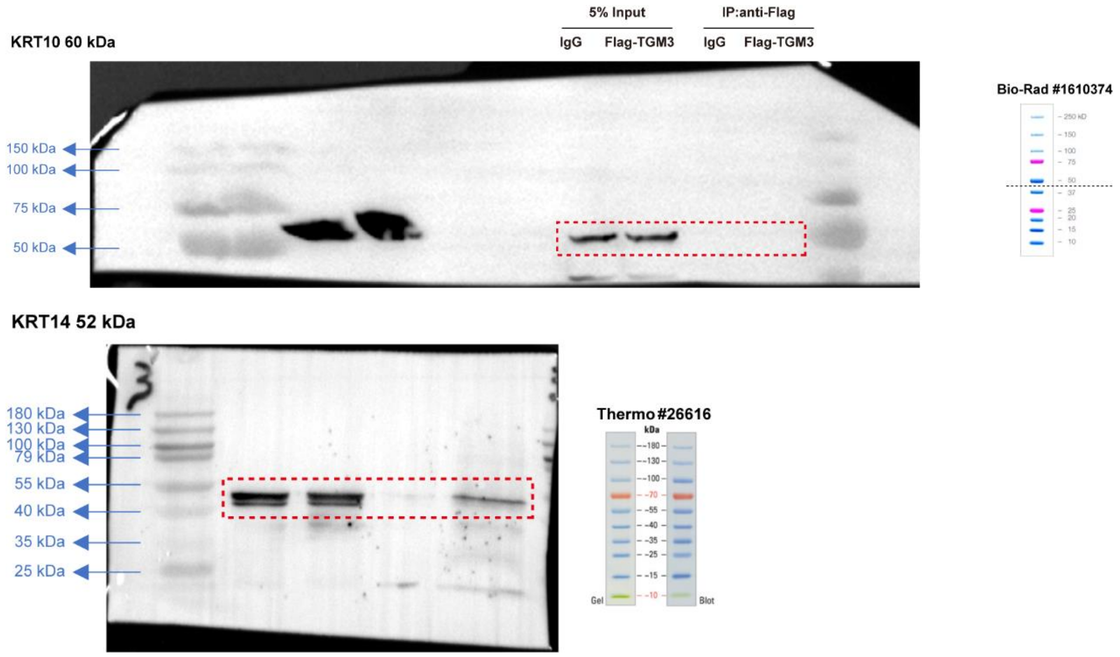
KRT10 60 kDa
5% Input
IP:anti-Flag
IgG
Flag-TGM3
IgG
Flag-TGM3
150 kDa
100 kDa
75 kDa
50 kDa
Bio-Rad #1610374
KRT14 52 kDa
180 kDa
130 kDa
100 kDa
79 kDa
55 kDa
40 kDa
35 kDa
25 kDa
Thermo #26616

Figure 3I

GAPDH 36 kDa

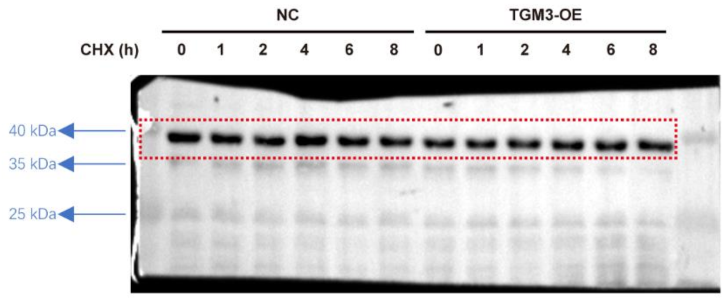

TGM3 72 kDa

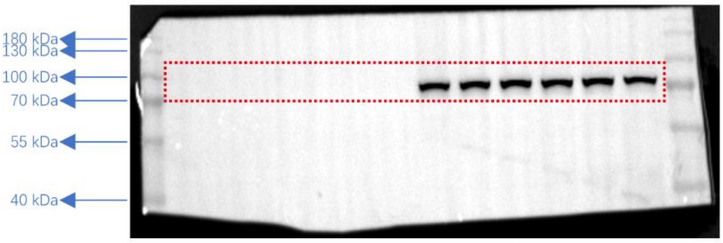

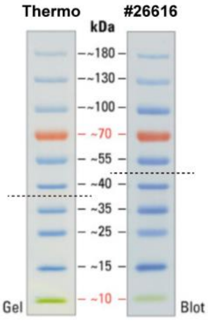

KRT14 52 kDa

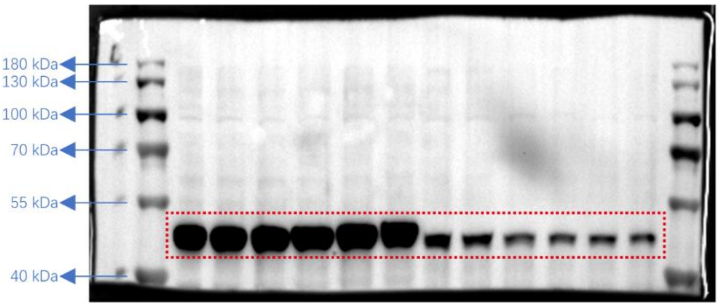

Figure 4D

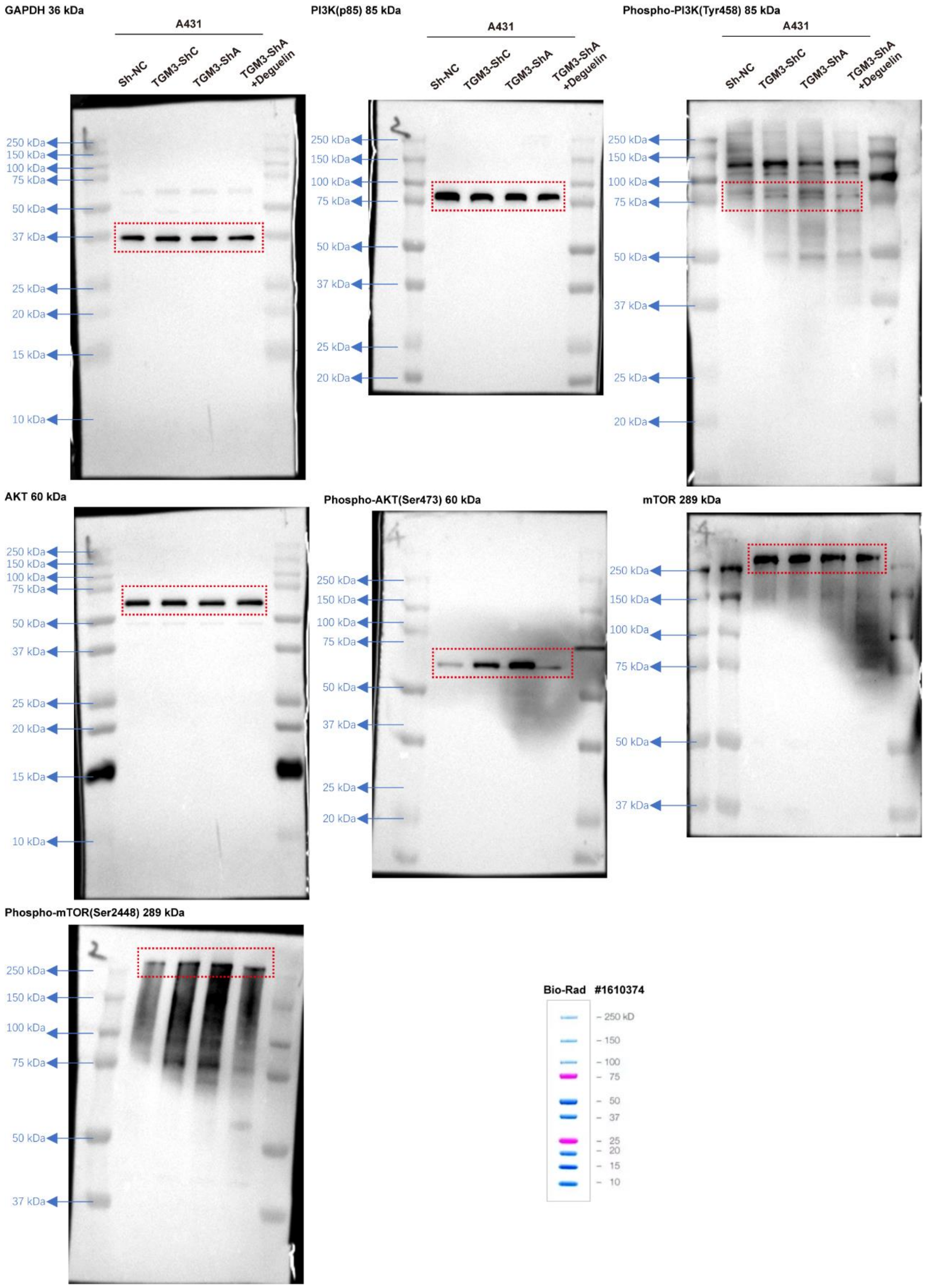
GAPDH 36 kDa
A431
Sh-NC
TGM3-ShC
TGM3-ShA
TGM3-ShA +Deguelin
250 kDa
150 kDa
100 kDa
75 kDa
50 kDa
37 kDa
25 kDa
20 kDa
15 kDa
10 kDa
PI3K(p85) 85 kDa
A431
Sh-NC
TGM3-ShC
TGM3-ShA
TGM3-ShA +Deguelin
250 kDa
150 kDa
100 kDa
75 kDa
50 kDa
37 kDa
25 kDa
20 kDa
Phospho-PI3K(Tyr458) 85 kDa
A431
Sh-NC
TGM3-ShC
TGM3-ShA
TGM3-ShA +Deguelin
250 kDa
150 kDa
100 kDa
75 kDa
50 kDa
37 kDa
25 kDa
20 kDa
AKT 60 kDa
250 kDa
150 kDa
100 kDa
75 kDa
50 kDa
37 kDa
25 kDa
20 kDa
15 kDa
10 kDa
Phospho-AKT(Ser473) 60 kDa
250 kDa
150 kDa
100 kDa
75 kDa
50 kDa
37 kDa
25 kDa
20 kDa
mTOR 289 kDa
250 kDa
150 kDa
100 kDa
75 kDa
50 kDa
37 kDa
Phospho-mTOR(Ser2448) 289 kDa
250 kDa
150 kDa
100 kDa
75 kDa
50 kDa
37 kDa
Bio-Rad #1610374
250 kD
150
100
75
50
37
25
20
15
10

Vimentin 54 kDa

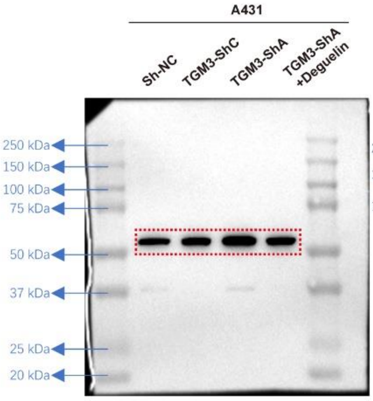

N-Cadherin 125 kDa

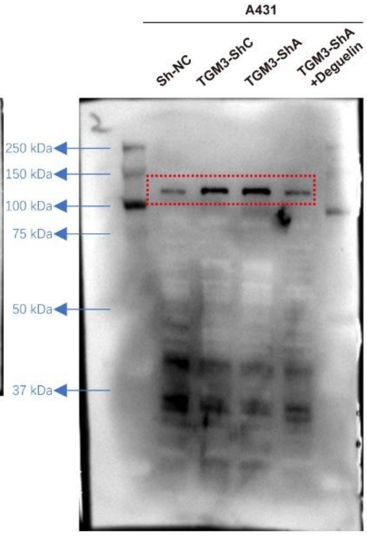

E-Cadherin 135 kDa

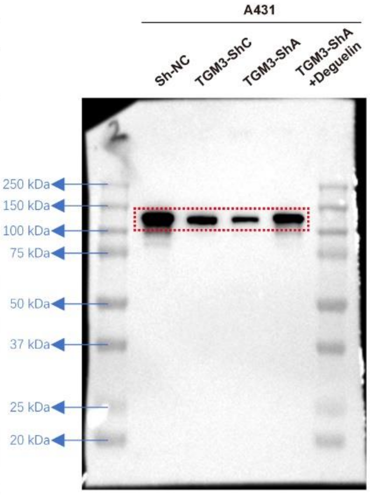

KRT14 52 kDa

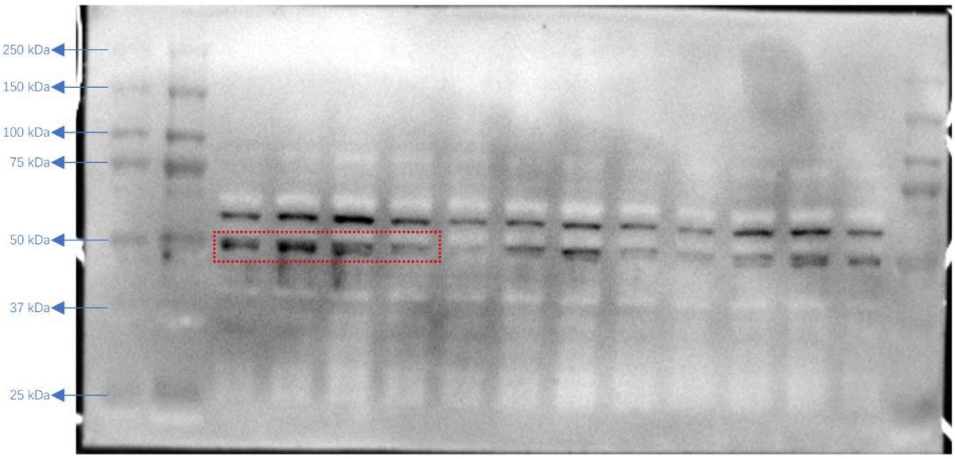

Bio-Rad #1610374

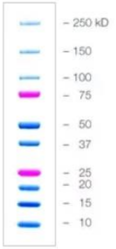

GAPDH 36 kDa

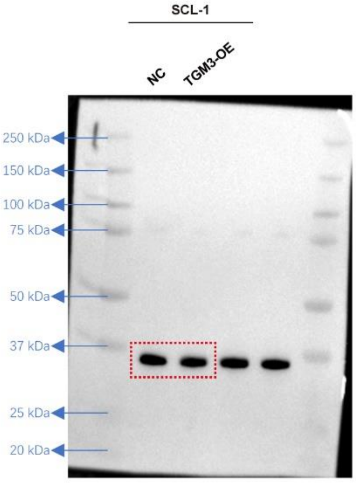

PI3K(p85) 85 kDa

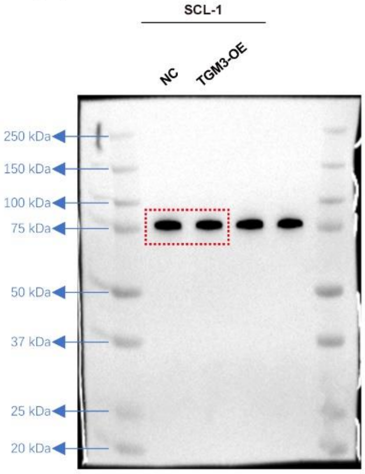

Phospho-PI3K(Tyr458) 85 kDa

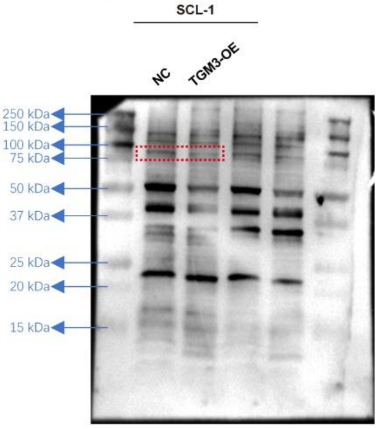

AKT 60 kDa

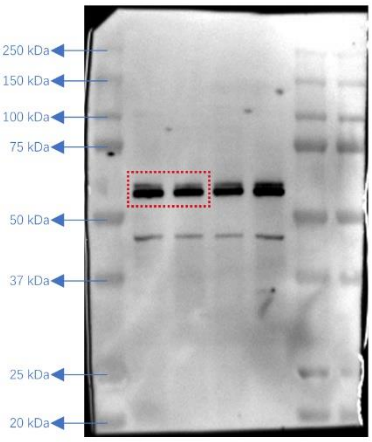

Phospho-AKT(Ser473) 60 kDa

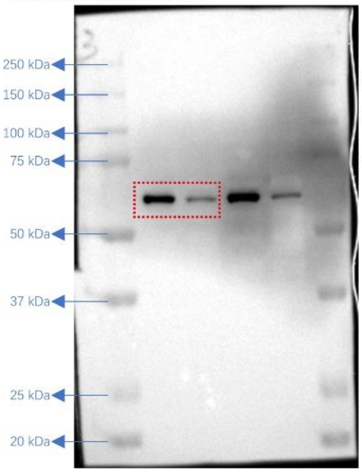

mTOR 289 kDa

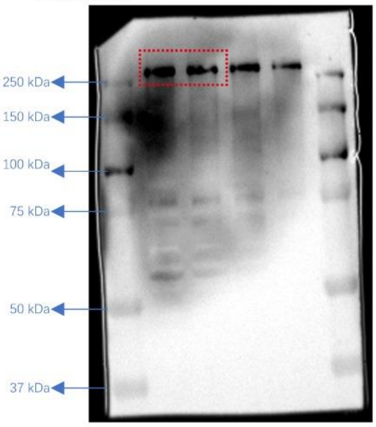

Phospho-mTOR(Ser2448) 289 kDa

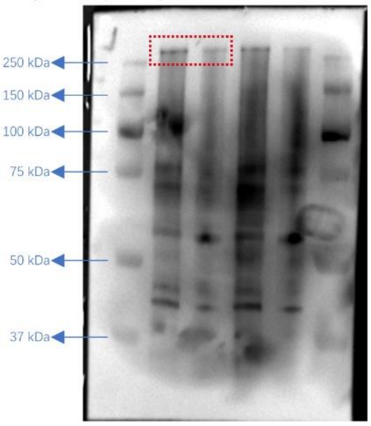

Bio-Rad #1610374

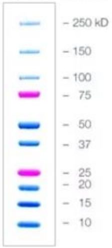

Vimentin 54 kDa

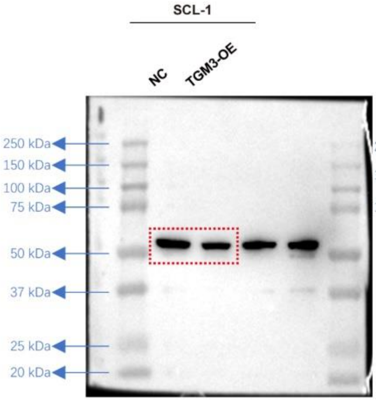

N-Cadherin 125 kDa

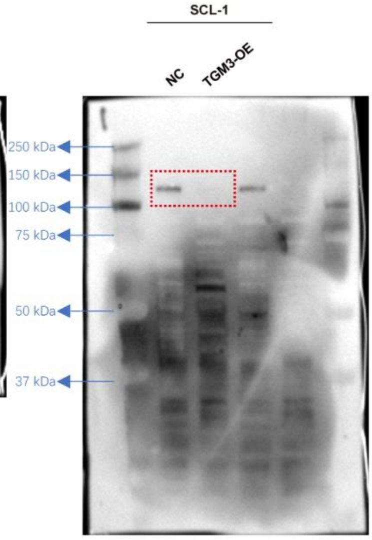

E-Cadherin 135 kDa

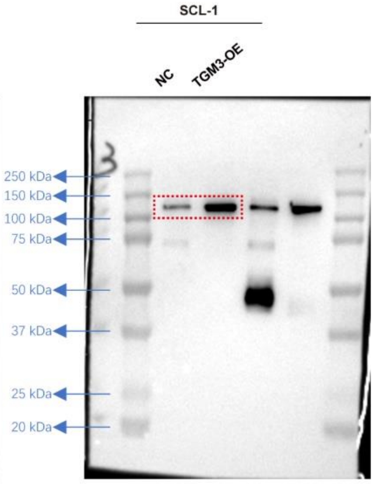

KRT14 52 kDa

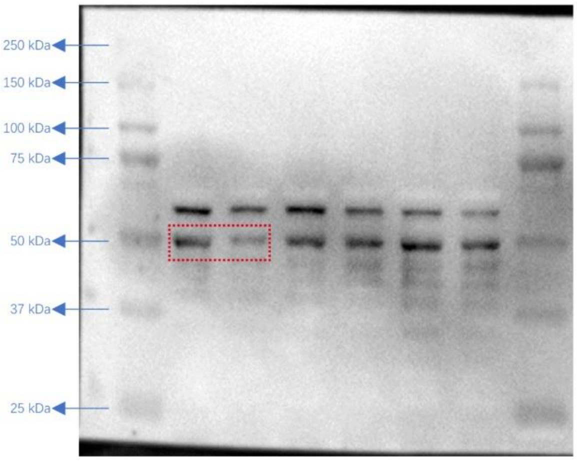

Bio-Rad #1610374

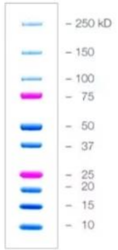

Figure 4E

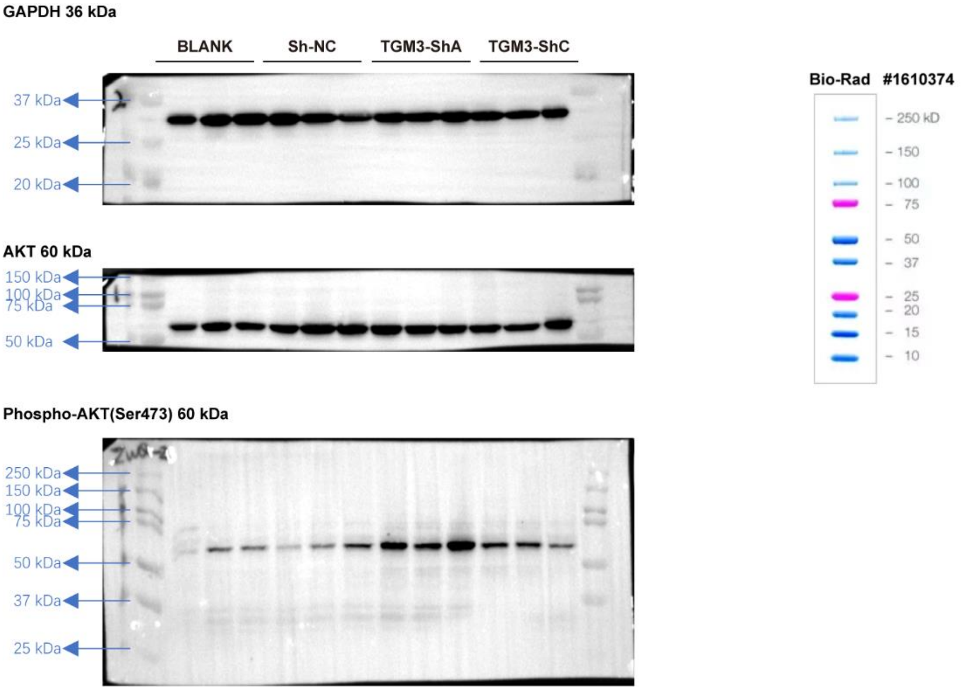
GAPDH 36 kDa
BLANK
Sh-NC
TGM3-ShA
TGM3-ShC
37 kDa
25 kDa
20 kDa
AKT 60 kDa
150 kDa
100 kDa
75 kDa
50 kDa
Phospho-AKT(Ser473) 60 kDa
250 kDa
150 kDa
100 kDa
75 kDa
50 kDa
37 kDa
25 kDa
Bio-Rad #1610374
250 kD
150
100
75
50
37
25
20
15
10

Figure 4F

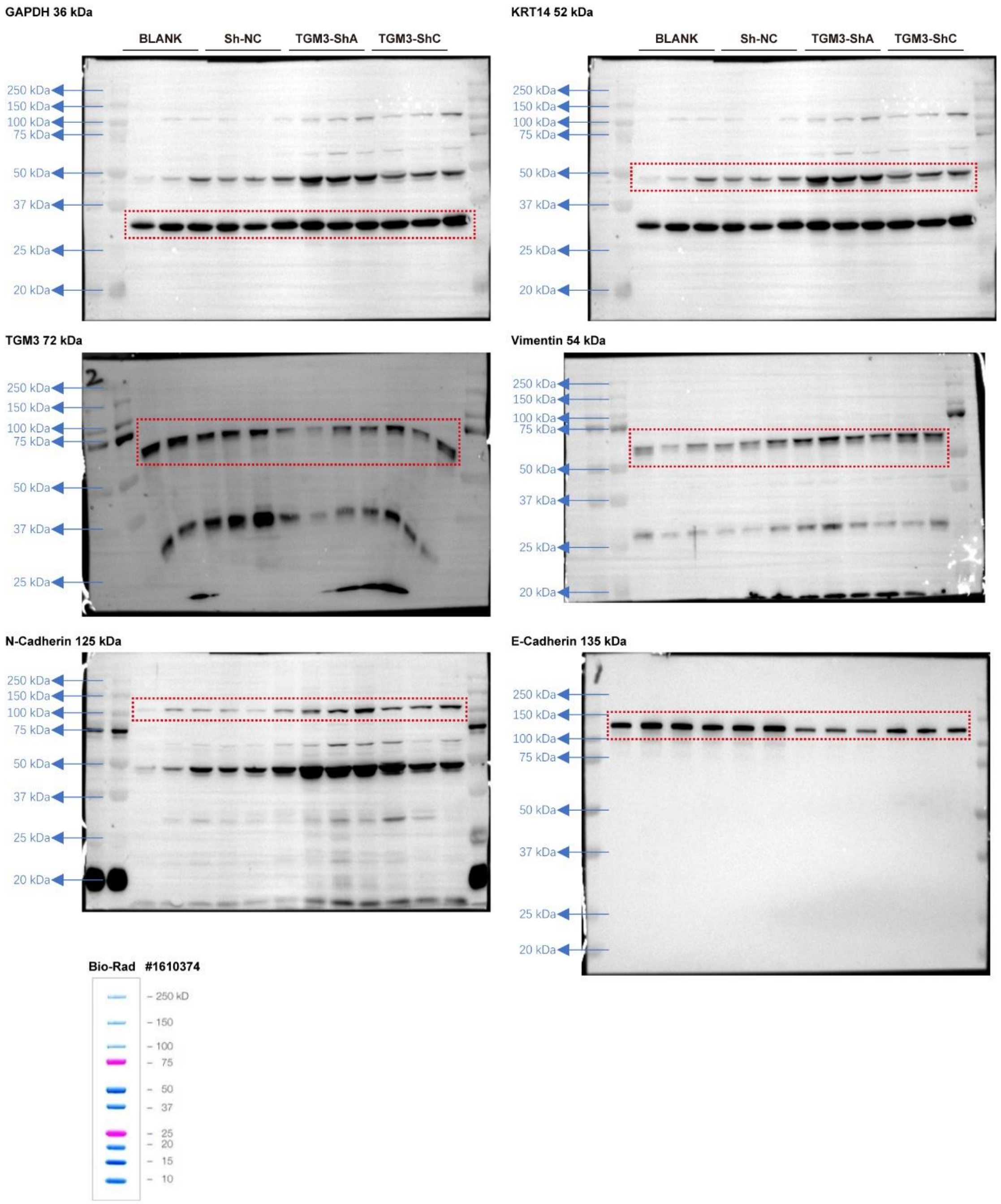
GAPDH 36 kDa
KRT14 52 kDa
BLANK
Sh-NC
TGM3-ShA
TGM3-ShC
TGM3 72 kDa
Vimentin 54 kDa
N-Cadherin 125 kDa
E-Cadherin 135 kDa
250 kDa
150 kDa
100 kDa
75 kDa
50 kDa
37 kDa
25 kDa
20 kDa
Bio-Rad #1610374
250 kD
150
100
75
50
37
25
20
15
10

Figure S1a

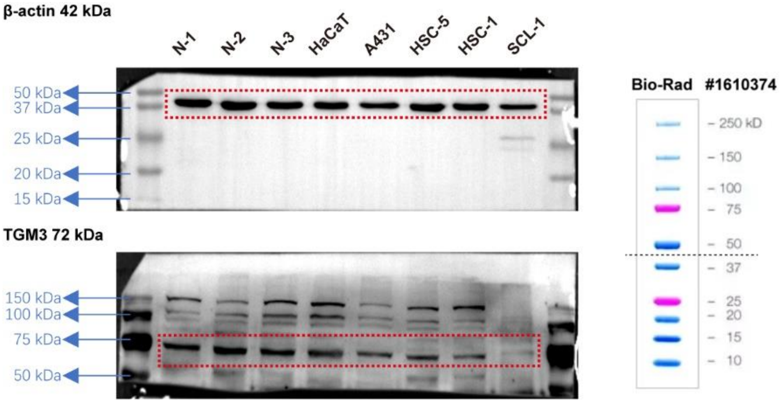

Figure S1b

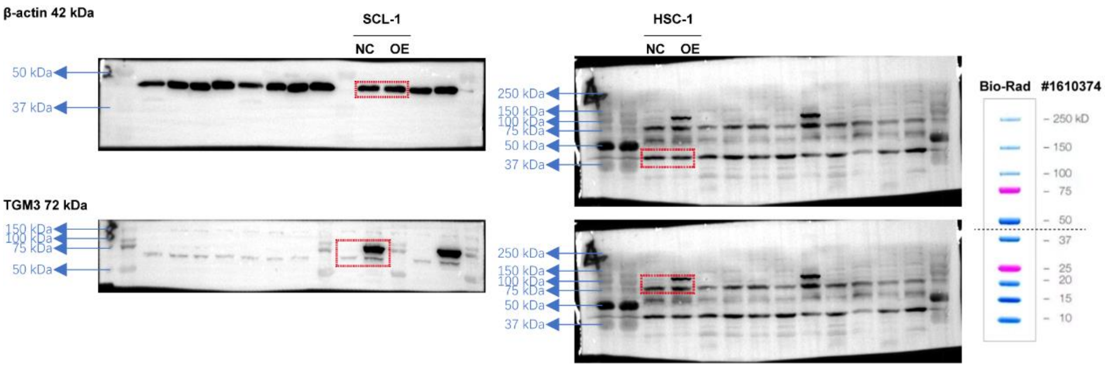

Figure S1c

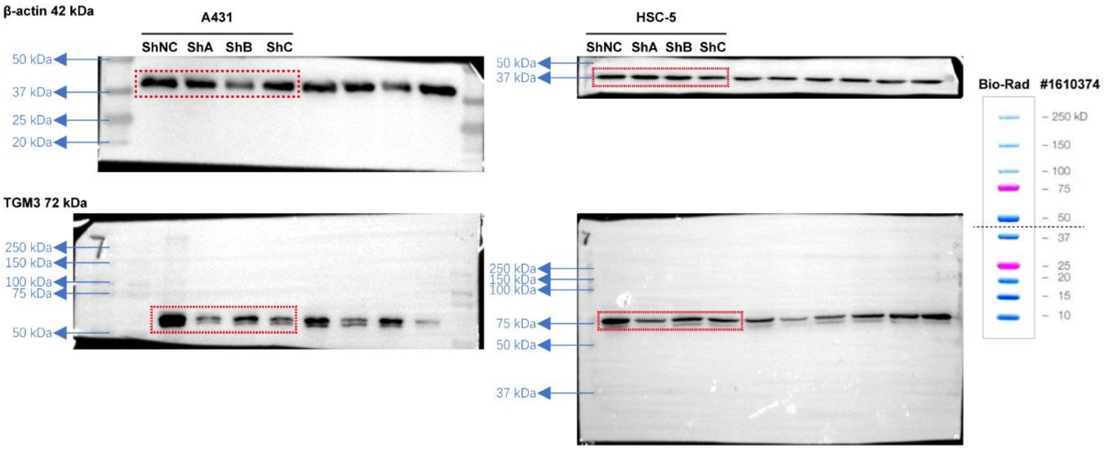

Figure S2a

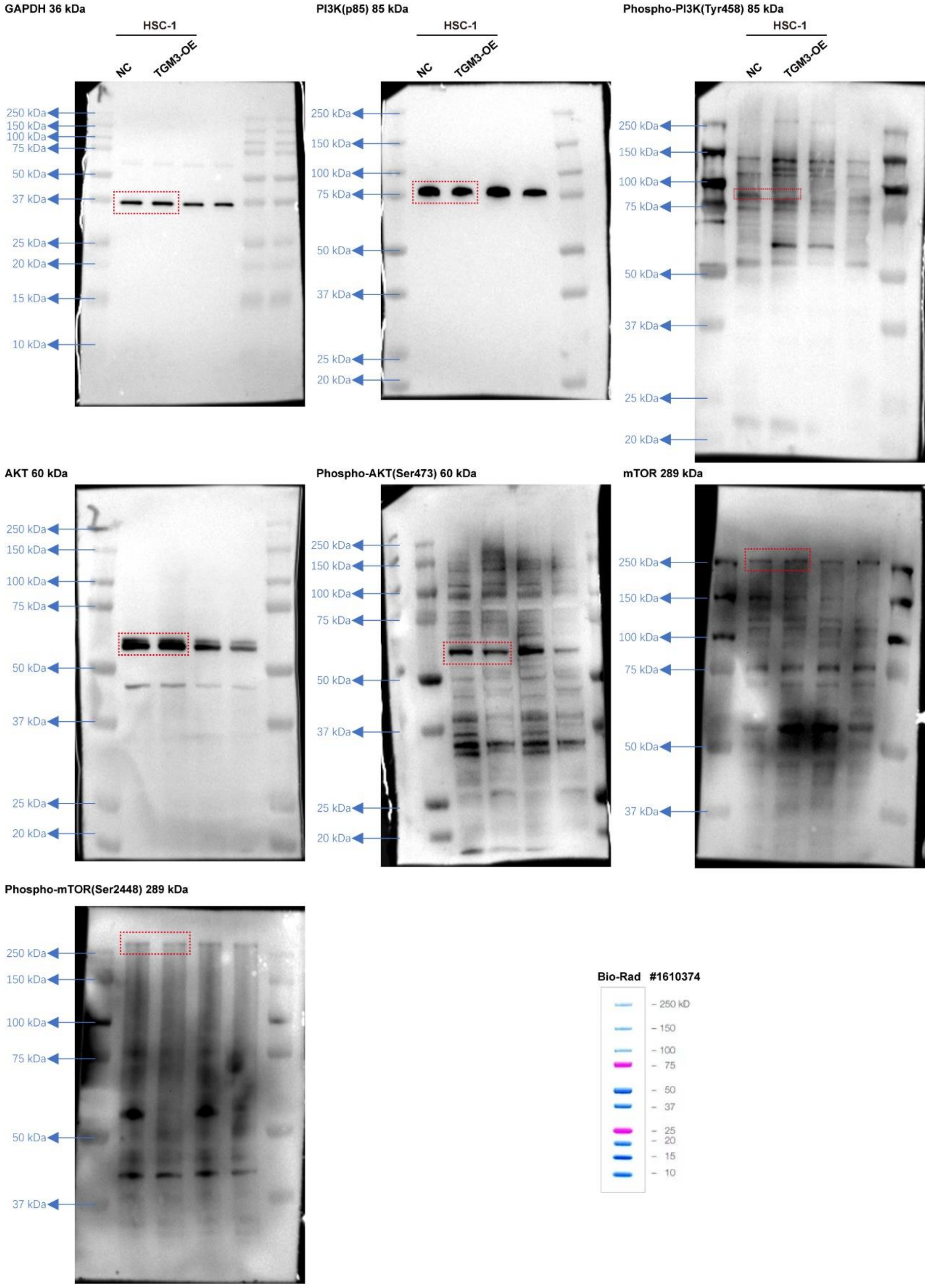

GAPDH 36 kDa
HSC-1
NC
TGM3-OE
250 kDa
150 kDa
100 kDa
75 kDa
50 kDa
37 kDa
25 kDa
20 kDa
15 kDa
10 kDa
PI3K(p85) 85 kDa
Phospho-PI3K(Tyr458) 85 kDa
AKT 60 kDa
Phospho-AKT(Ser473) 60 kDa
mTOR 289 kDa
Phospho-mTOR(Ser2448) 289 kDa
Bio-Rad #1610374
250 kD
150
100
75
50
37
25
20
15
10

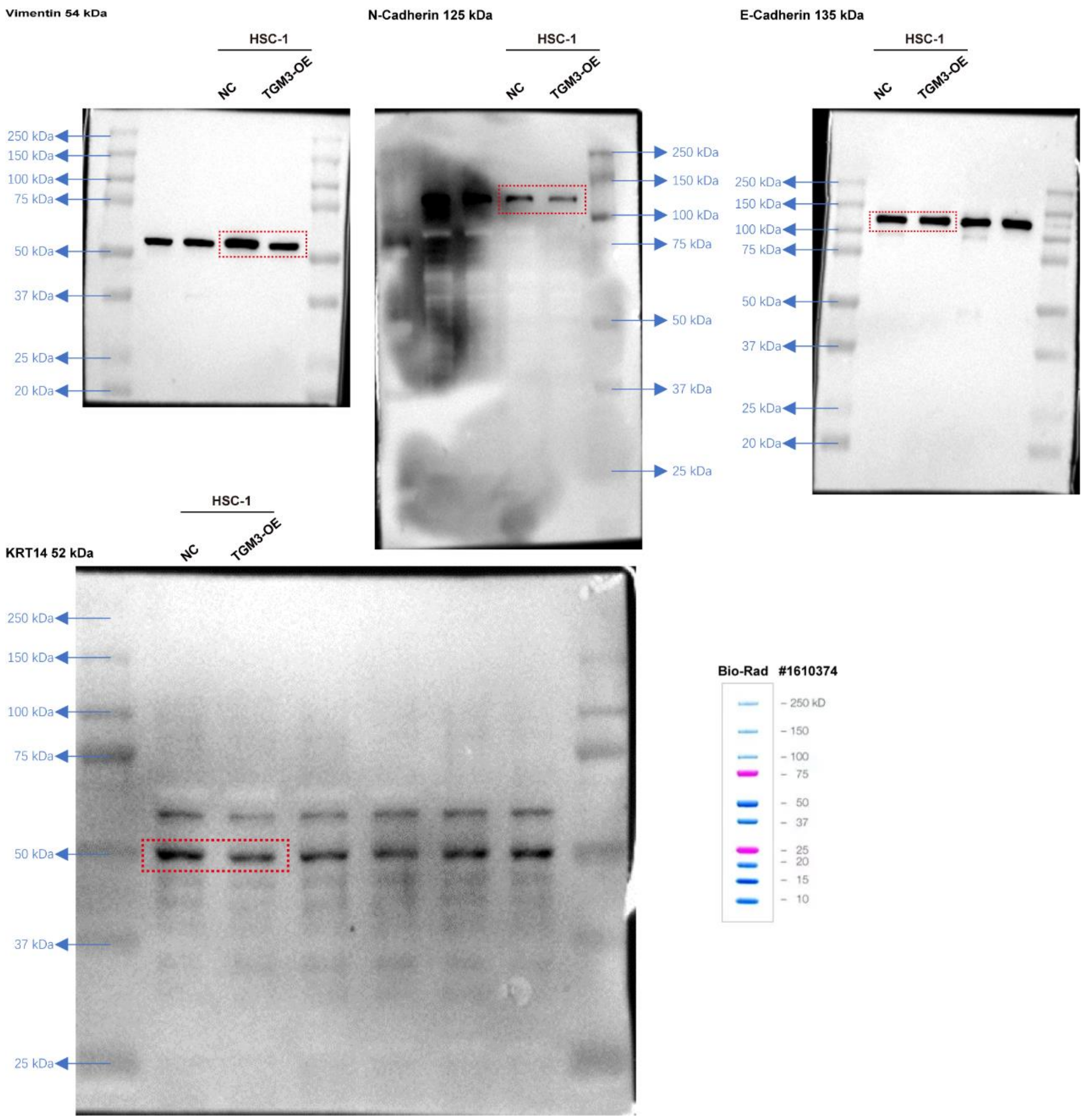

Vimentin 54 kDa
HSC-1
NC
TGM3-OE
250 kDa
150 kDa
100 kDa
75 kDa
50 kDa
37 kDa
25 kDa
20 kDa
N-Cadherin 125 kDa
HSC-1
NC
TGM3-OE
250 kDa
150 kDa
100 kDa
75 kDa
50 kDa
37 kDa
25 kDa
E-Cadherin 135 kDa
HSC-1
NC
TGM3-OE
250 kDa
150 kDa
100 kDa
75 kDa
50 kDa
37 kDa
25 kDa
20 kDa
KRT14 52 kDa
HSC-1
NC
TGM3-OE
250 kDa
150 kDa
100 kDa
75 kDa
50 kDa
37 kDa
25 kDa
Bio-Rad #1610374
250 kD
150
100
75
50
37
25
20
15
10

Figure S2b

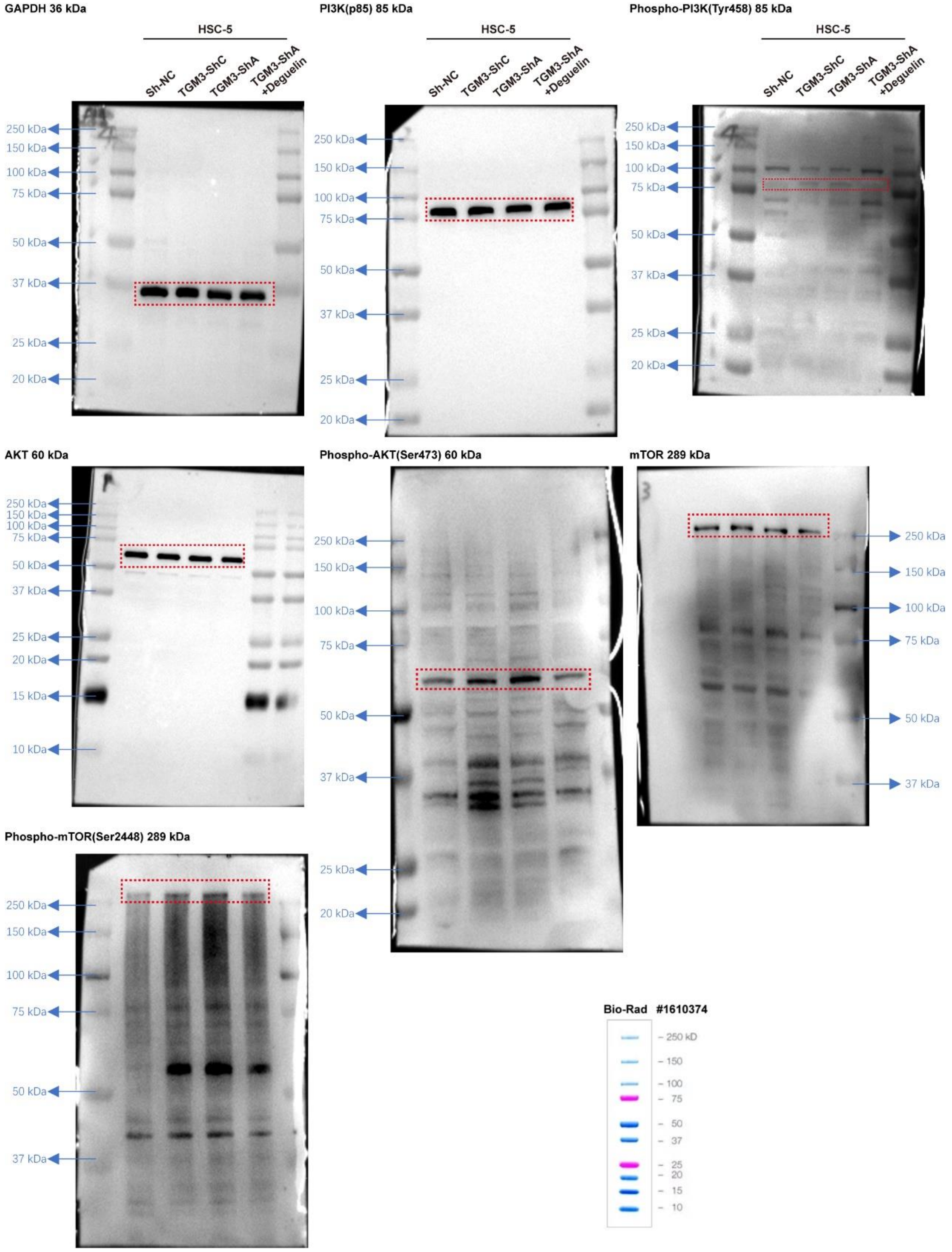
GAPDH 36 kDa
PI3K(p85) 85 kDa
Phospho-PI3K(Tyr458) 85 kDa
HSC-5
Sh-NC
TGM3-ShC
TGM3-ShA
TGM3-ShA +Deguelin
250 kDa
150 kDa
100 kDa
75 kDa
50 kDa
37 kDa
25 kDa
20 kDa
15 kDa
10 kDa
AKT 60 kDa
Phospho-AKT(Ser473) 60 kDa
mTOR 289 kDa
Phospho-mTOR(Ser2448) 289 kDa
Bio-Rad #1610374
250 kD
150
100
75
50
37
25
20
15
10

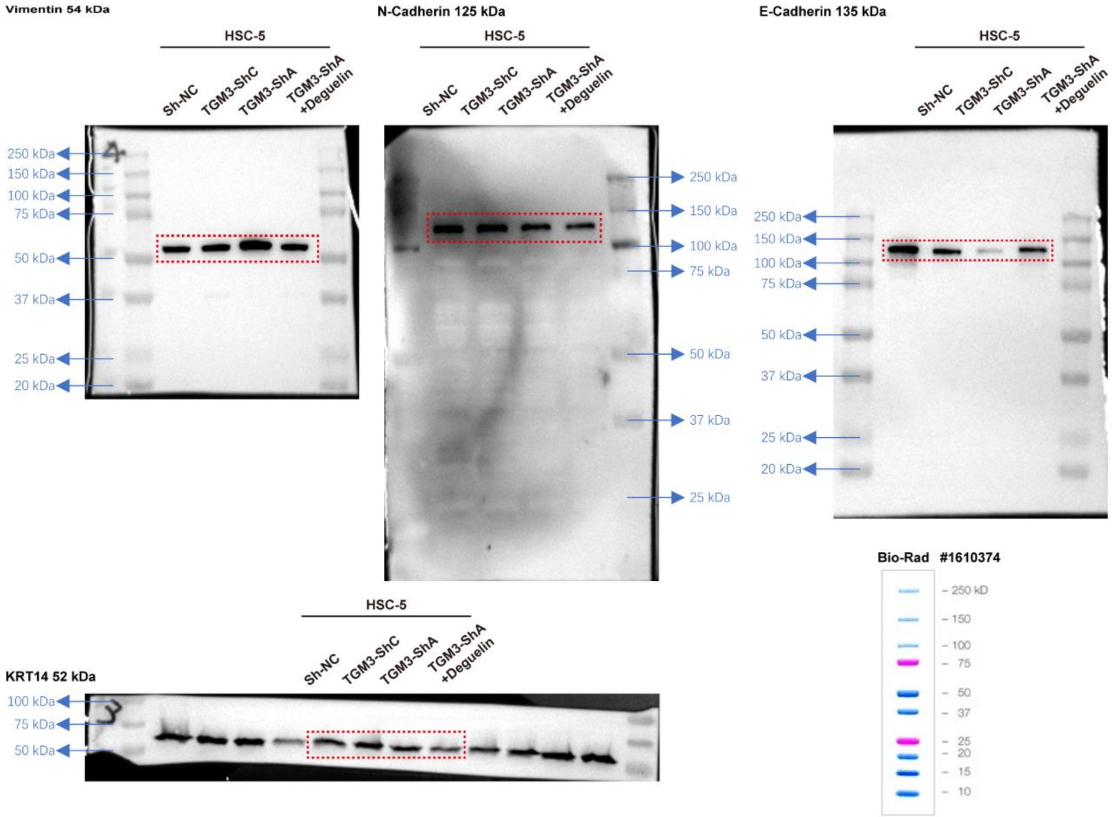

Vimentin 54 kDa
HSC-5
Sh-NC
TGM3-ShC
TGM3-ShA
TGM3-ShA +Deguelin
250 kDa
150 kDa
100 kDa
75 kDa
50 kDa
37 kDa
25 kDa
20 kDa
N-Cadherin 125 kDa
HSC-5
Sh-NC
TGM3-ShC
TGM3-ShA
TGM3-ShA +Deguelin
250 kDa
150 kDa
100 kDa
75 kDa
50 kDa
37 kDa
25 kDa
E-Cadherin 135 kDa
HSC-5
Sh-NC
TGM3-ShC
TGM3-ShA
TGM3-ShA +Deguelin
250 kDa
150 kDa
100 kDa
75 kDa
50 kDa
37 kDa
25 kDa
20 kDa
Bio-Rad #1610374
250 kD
150
100
75
50
37
25
20
15
10
KRT14 52 kDa
HSC-5
Sh-NC
TGM3-ShC
TGM3-ShA
TGM3-ShA +Deguelin
100 kDa
75 kDa
50 kDa
